# Perineural invasion affects prognosis of patients undergoing colorectal cancer surgery: a propensity score matching analysis

**DOI:** 10.1186/s12885-023-10936-w

**Published:** 2023-05-18

**Authors:** Le Qin, Yixin Heng, Shenghe Deng, Junnan Gu, Fuwei Mao, Yifan Xue, Zhenxing Jiang, Jun Wang, Denglong Cheng, Ke Wu, Yinghao Cao, Kailin Cai

**Affiliations:** 1 Department of Gastrointestinal Surgery, Union Hospital, Tongji Medical College, Huazhong University of Science and Technology, Wuhan, 430022 Hubei China; 2 Department of General Surgery, The First Affiliated Hospital of Shihezi University, Shihezi, 832008 Xinjiang P.R. China; 3 Department of Digestive Surgical Oncology, Cancer Center, Union Hospital, Tongji Medical College, Huazhong University of Science and Technology, Wuhan, 430022 China; 4 Cancer Center, Union Hospital, Tongji Medical College, Huazhong University of Science and Technology, Wuhan, China

**Keywords:** Colorectal cancer, Perineural invasion, Propensity score matching, Overall survival, Disease-free survival

## Abstract

**Background:**

Tumour perineural invasion (PNI) is a predictor of poor prognosis, but its effect on the prognosis of patients with colorectal cancer (CRC) has not yet been elucidated.

**Methods:**

This retrospective study used propensity score matching (PSM). The clinical case data of 1470 patients with surgically treated stage I–IV CRC at Wuhan Union Hospital were collected. PSM was used to analyse and compare the clinicopathological characteristics, perioperative outcomes, and long-term prognostic outcomes of the PNI(+) and PNI(-) groups. The factors influencing prognosis were screened using Cox univariate and multivariate analyses.

**Results:**

After PSM, 548 patients were included in the study (n = 274 in each group). Multifactorial analysis showed that neurological invasion was an independent prognostic factor affecting patients’ OS and DFS (hazard ratio [HR], 1.881; 95% confidence interval [CI], 1.35–2.62; P = 0.0001; HR, 1.809; 95% CI, 1.353–2.419; P < 0.001). Compared to PNI(+) patients without chemotherapy, those who received chemotherapy had a significant improvement in OS (P < 0.01). The AUROC curve of OS in the PNI(+) subgroup (0.802) was higher than that after PSM (0.743), while that of DFS in the PNI(+) subgroup (0.746) was higher than that after PSM (0.706). The independent predictors of PNI(+) could better predict the prognosis and survival of patients with PNI(+).

**Conclusions:**

PNI significantly affects the long-term survival and prognosis of patients with CRC undergoing surgery and is an independent risk factor for OS and DFS in patients with CRC undergoing surgery. Postoperative chemotherapy significantly improved the OS of PNI(+) patients.

**Supplementary Information:**

The online version contains supplementary material available at 10.1186/s12885-023-10936-w.

## Background

Colorectal cancer (CRC) is among the most common malignant tumours. According to the Global Cancer Statistics report, the incidence of CRC ranks third after breast cancer and lung cancer (10.0%), while the mortality rate ranks second (9.4%)[1, 2]. With the continuous improvement and development of anatomical, molecular biology, and postoperative pathological examinations, the perineural invasion (PNI) of CRC has gained increasing researcher attention. PNI was first reported in the European literature by scientists who described a tendency for head and neck tumours to grow along nerves towards the cranial fossa [3]. Since then, PNI has been identified as a key pathological feature of many other malignancies, including tumours of the pancreas, colon, rectum, prostate, biliary tract, and stomach, for which it is a marker of poor prognosis and reduced survival [4, 5]. The incidence of PNI is higher in pancreatic cancer (98%), bile duct cancer (75–85%), prostate cancer (75%), and gastric adenocarcinoma (60%); however, in CRC, the incidence of PNI seems much lower, only 20–33%[5]. Therefore, PNI is not only an important indicator of benign and malignant tumours, it is useful for tumour staging [6].

Tumour invasion and metastasis are important contributors to a poor prognosis and shortened survival in patients with CRC. PNI is defined as tumour growth in, around, and through nerves and nerve sheaths, implying that it is more aggressive. The tumours of stage II patients may not have metastasised to the lymph nodes; however, they may have invaded the nerves and created vascular thrombi. Although PNI is a sign of poor survival in CRC, general consensus is lacking on its usefulness in staging and treatment decisions. The results of relevant studies are different and even contradictory. Han et al. [7] reported that the occurrence of tumour PNI was not related to age, sex, and distant tumour metastasis but was closely related to tumour size, location, depth of invasion, vascular invasion, and lymph node metastasis and that the prognosis was significantly worse for PNI(+) than PNI(-) patients. Li et al. [8] and Kaya et al. [9] also reported that PNI is a risk factor affecting the prognosis of patients. However, Hu G et al. [10] reported that PNI was not an independent poor prognostic factor for CRC patients and that PNI patients may not benefit from postoperative adjuvant chemotherapy. This may be closely related to the lack of bias control and small sample size of these studies. The differences in study design create controversy regarding prognostic factors, creating the need for a larger sample size to support the relevant conclusions.

Therefore, here we collected data from a large sample database from a single centre and used PSM to thoroughly explore the effect of PNI on short-term outcomes, tumour recurrence, and long-term survival of patients with surgically treated CRC. It can not only reduce the influence of covariates on the research results but also effectively reduce various biases described below.

## Methods

### General date

The data of 1470 patients with CRC who underwent resection in Wuhan Union Hospital from February 2014 to May 2018 were retrospectively analysed. In this study, cases of stage I–III CRC were treated with colorectal resection plus en bloc regional lymph node dissection [11, 12], while those of stage IV CRC were treated with simultaneous or staged metastasectomy. The inclusion criteria were as follows: (1) age > 18 years with CRC confirmed by imaging or pathology (Union for International Cancer Control CRC Staging Manual, 7th edition); (2) underwent resection of primary CRC for the first time; (3) availability of complete clinical and pathological data; and (4) preoperative with or without nerve invasion. The exclusion criteria were as follows: (1) preoperative neoadjuvant therapy; (2) availability of incomplete clinical and pathological data; and (3) presence of other tumours. PNI positivity was defined as tumour cells invading any layer of the nerve sheath or growing along more than 1/3 of the nerve perimeter after haematoxylin-eosin staining.

### Data collection

Collected data included sex, age, body mass index (BMI), smoking history, family history of cancer, history of previous abdominal surgery, neoadjuvant chemotherapy, complications (cardiovascular disease, cerebrovascular disease, chronic obstructive pulmonary disease, diabetes), maximum tumour diameter, tumour location, history of preoperative obstruction, postoperative radiotherapy, postoperative chemotherapy, vascular tumour thrombus, degree of differentiation, T stage, N stage, M stage, and *KRAS* mutation status. Perioperative data included American Anaesthesiologist (ASA) score, surgical method, requirement for blood transfusion, primary anastomosis, perineal tamponade, postoperative complications (postoperative intestinal obstruction, anastomotic fistula, surgical area infection, and cardiovascular and cerebrovascular diseases), length of stay, and preoperative tumour markers (carcinoembryonic antigen [CEA], carbohydrate antigen 19 − 9 [CA 19 − 9], and cancer antigen 125 [CA125]).

### Follow-up

We established a standard follow-up process according to the international consensus on CRC management. Starting the day after surgery, patients were followed up by phone or at the hospital. Patients were followed up every 3 months for the first 2 years after surgery and every 6 months from years 3 to 5. Patients who failed to attend an appointment within 1 year of their last visit were considered lost to follow-up. The follow-up information collected included adjuvant therapy (radiotherapy or chemotherapy) and the presence of tumour recurrence; if recurrence occurred, the time to recurrence, survival time, and timing of death were recorded. The latest follow-up date of this study was August 2021. Overall survival (OS) was defined as the time from surgery to death or the end of follow-up. Disease-free survival (DFS) was defined as the time from the day of surgery to tumour recurrence, metastasis, or the end of follow-up. The study was approved by the Ethics Committee of Wuhan Union Hospital, and all methods were performed in accordance with the relevant guidelines and regulations. All procedures in this study involving human participants were performed in accordance with the Declaration of Helsinki.

### Propensity score matching

**Fig. 1:**
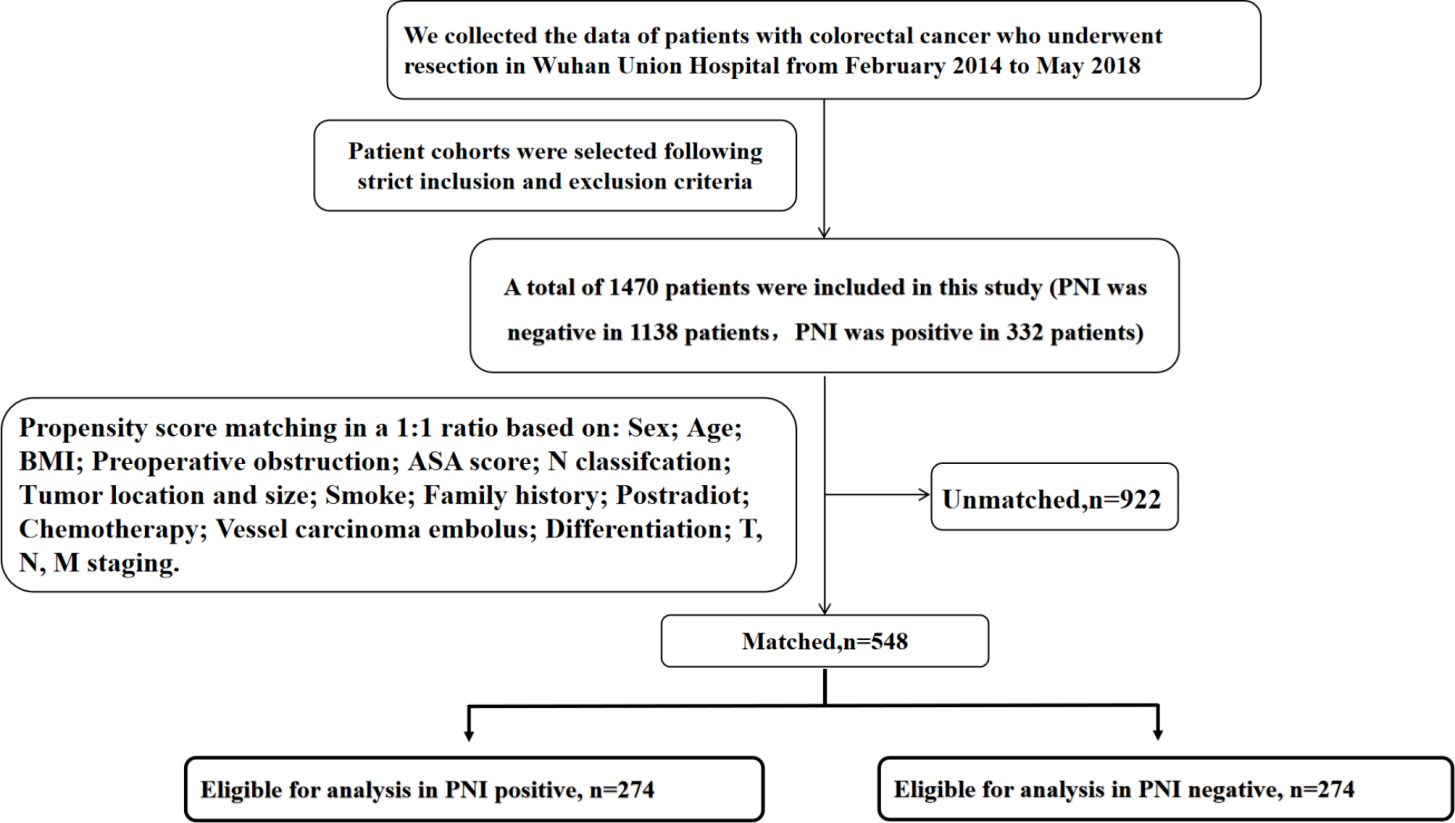
Strategies for patient selection in the study

Based on the PNI results, 1:1 propensity score matching (PSM) without replacement and a matching tolerance of 0.01 was performed using the covariates including sex, age, BMI, maximum straight diameter of tumour, tumour location, history of preoperative intestinal obstruction, smoking history, family history of tumour, postoperative radiotherapy, postoperative chemotherapy, vascular tumour thrombus, degree of differentiation, and tumour node metastasis (TNM) stage. From our database of 1470 patients, matching yielded 548 patients who met the inclusion criteria (Fig. 1).

### Statistical methods

All statistical analyses were performed using SPSS statistical software (SPSS 25.0, Chicago, IL,USA). Count data were expressed as percentage, and comparison between groups was performed using the chi-square test or Fisher exact probability method. Continuous variables were expressed as median and quartile, and comparison between groups was performed using the Mann-Whitney U test. Kaplan-Meier survival curve was used to describe OS and DFS, and Log-rank test was used for comparison between the two groups. Univariate analysis showed that the indicators related to survival (P < 0.05) performed multivariate Cox regression analysis and calculated hazard ratios (HRs) and corresponding 95% confidence intervals (CIs). P < 0.05 was considered statistically significant.

## Results

### Patients’ demographic characteristics and clinicopathological features

Our results showed that 1138 patients (77.40%) were in the PNI(-) group versus 332 patients (22.60%) in the PNI(+) group. Compared with patients with PNI(-) status, patients with PNI(+) status had a larger average tumour diameter (87.90% vs. 80.20%; P < 0.001), increased preoperative intestinal obstruction and vascular invasion (21.70% vs. 11.10%, P < 0.001; 45.80% vs. 10.60%, P < 0.001), and the proportion of patients receiving postoperative chemotherapy was significantly increased (60.80% vs. 50.70%, P = 0.001). In the PNI(+) group, the proportion of patients with moderate differentiation was higher (74.40% vs. 69.40%, P = 0.008), while the proportion of patients with stage III or IV disease was significantly increased (73.20% vs. 41.30%; P < 0.001); CEA (47.30% vs. 36.60%; P < 0.001), CA 19 − 9 (25.00% vs. 15.30%; P < 0.001), and CA125 (14.50% vs. 9.30%; P = 0.007) were significantly higher, with statistically significant differences. There was no significant intergroup difference in sex, age, BMI, smoking history, tumour family history, tumour location, ASA score, previous abdominal surgery history, preoperative new aids, cardiovascular disease, cerebrovascular disease, chronic obstructive pulmonary disease, diabetes, or complications (P > 0.05) (Supplementary Table 1). After PSM, there were 274 people in the PNI(+) group and PNI(-) group after PSM and no significant difference between PNI status and clinical pathological characteristics (P > 0.05) (Table 1).

**Table 1:** Characteristics of patients in the post-matching cohort

Characteristics		PNI(-) (N = 274)	PNI(+) (N = 274)	Z/X^2^	P
**PSM score**		0.33 ± 0.20	0.33 ± 0.20	-0.009	0.993
**Age (years)**				0.030	0.863
	**≤ 60**	156 (56.90%)	154 (56.20%)		
	**>60**	118 (43.10%)	120 (43.80%)		
**BMI**		22.90 (21.30-24.49)	22.66 (20.83–24.24)	-1.268	0.205
**Tumor size (cm)**				2.969	0.227
	**≤ 2.70**	36 (13.10%)	32 (11.70%)		
	**2.70–4.40**	110 (40.10%)	130 (47.40%)		
	**>4.40**	128 (46.70%)	112 (40.90%)		
**Obstruction before surgery**				0.117	0.733
	**Absent**	229 (83.60%)	226 (82.50%)		
	**present**	45 (16.40%)	48 (17.50%)		
**Sex**				0.070	0.792
	**Male**	172 (62.80%)	169 (61.70%)		
	**Female**	102 (37.20%)	105 (38.30%)		
**Family history of cancer**				0.171	0.679
	**No**	31 (11.30%)	28 (10.20%)		
	**Yes**	243 (88.70%)	246 (89.80%)		
**Post radiotherapy**				1.129	0.288
	**No**	260 (94.90%)	254 (92.70%)		
	**Yes**	14 (5.10%)	20 (7.30%)		
**Adjuvant chemotherapy**				1.072	0.301
	**No**	124 (45.30%)	112 (40.90%)		
	**Yes**	150 (54.70%)	162 (59.10%)		
**Vascular cancer embolus**				1.185	0.276
	**Absent**	189 (69.00%)	177 (64.60%)		
	**Present**	85 (31.00%)	97 (35.40%)		
**Histological grade**				-0.801	0.423
	**Poorly**	56 (20.40%)	41 (15.00%)		
	**Moderately**	187 (68.20%)	208 (75.90%)		
	**Well**	31 (11.30%)	25 (9.10%)		
**Stage**				-0.222	0.825
	**I**	5 (1.80%)	7 (2.60%)		
	**II**	83 (30.30%)	76 (27.70%)		
	**III**	138 (50.40%)	152 (55.50%)		
	**IV**	48 (17.50%)	39 (14.20%)		
**T stage**				-1.096	0.273
	**T1**	1 (0.40%)	5 (1.80%)		
	**T2**	13 (4.70%)	12 (4.40%)		
	**T3**	167 (60.90%)	175 (63.90%)		
	**T4**	93 (33.90%)	82 (29.90%)		
**N stage**				-0.417	0.677
	**N0**	105 (38.30%)	102 (37.20%)		
	**N1**	87 (31.80%)	102 (37.20%)		
	**N2**	82 (29.90%)	70 (25.50%)		
**M stage**				-1.337	0.181
	**M0**	229 (83.60%)	240 (87.60%)		
	**M1**	45 (16.40%)	34 (12.40%)		
**Primary tumor location**				0.385	0.825
	**Right colon**	63 (23.00%)	65 (23.70%)		
	**Left colon**	67 (24.50%)	72 (26.30%)		
	**Rectum**	144 (52.60%)	137 (50.00%)		
**ASA**				6.472	0.087
	**1**	1 (0.40%)	7 (2.60%)		
	**2**	198 (72.30%)	190 (69.30%)		
	**3**	59 (21.50%)	53 (19.30%)		
	**4**	16 (5.80%)	24 (8.80%)		
**Previous history of abdominal surgery**				0.415	0.519
	**No**	217 (79.20%)	223 (81.40%)		
	**Yes**	57 (20.80%)	51 (18.60%)		
**Neoadjuvant chemotherapy**				2.409	0.121
	**No**	264 (96.40%)	256 (93.40%)		
	**Yes**	10 (3.60%)	18 (6.60%)		
**preoperative comorbidities**					
**Total patients**				3.067	0.080
	**No**	188 (68.60%)	202 (73.70%)		
	**Yes**	86 (31.40%)	72 (26.30%)		
**Cardiovascular disease**				3.233	0.072
	**No**	199 (72.60%)	217 (79.20%)		
	**Yes**	75 (27.40%)	57 (20.80%)		
**Cerebrovascular disease**				2.639	0.104
	**No**	264 (96.40%)	270 (98.50%)		
	**Yes**	10 (3.60%)	4 (1.50%)		
**COPD**				3.067	0.080
	**No**	271 (98.90%)	265 (96.70%)		
	**Yes**	3 (1.10%)	9 (3.30%)		
**Diabetes**				0.029	0.865
	**No**	255 (93.10%)	256 (93.40%)		
	**Yes**	19 (6.90%)	18 (6.60%)		
**CEA (ng/mL)**				0.359	0.549
	**< 5**	149 (54.40%)	142 (51.80%)		
	**≥ 5**	125 (45.60%)	132 (48.20%)		
**CA199 (kU/L)**				1.189	0.275
	**< 37**	212 (77.40%)	201 (73.40%)		
	**≥ 37**	62 (22.60%)	73 (26.60%)		
**CA125 (U/mL)**				0.069	0.793
	**< 35**	240 (87.60%)	242 (88.30%)		
	**≥ 35**	34 (12.40%)	32 (11.70%)		

Abbreviations: BMI, body mass index (calculated as weight in kilograms divided by height in meters squared); ASA, American Society of Anesthesiologists Physical Status Classification; COPD, chronic obstructive pulmonary disease; CEA, carcino-embryonic antigen; CA19-9; CA12-5, carbohydrate antigen. P values considered statistically significant are presented in bold

### Comparison of patients’ perioperative outcomes and long-term prognosis

Compared with the PNI(+) group, the PNI(-) group had a significantly longer postoperative hospital stay (median, 13 (interquartile range [IQR], 11–17) days vs. 13 (IQR, 10–15) days; P *=* 0.003). There was no significant correlation between PNI status and intraoperative management (surgical method, blood transfusion, primary anastomosis, perineal packing) or postoperative complications (intestinal obstruction, anastomotic leakage, surgical site infection, or cardiovascular and cerebrovascular diseases) (P > 0.05) (Supplementary Table 2). After PSM, except for length of stay (median, 14 [IQR, 11–18] days vs. 13 [IQR, 10–15] days; P < 0.001) and surgical method (laparoscopy: 44.50% vs. 52.90%; laparotomy: 55.50% vs. 47.10%; P = 0.049), no significant intergroup difference was noted *(*P *>* 0.05) (Table 2).

**Table 2:** Intraoperative management and postoperative complication outcomes in the post-matching cohort

Characteristics		PNI(-) (N = 274)	PNI(+) (N = 274)	Z/X^2^	P
Intraoperative management					
**Type of surgery**		122 (44.50%)	145 (52.90%)	3.864	**0.049**
	**Laparoscopic**	152 (55.50%)	129 (47.10%)		
	**Laparotomy**				
**Blood transfusion**		208 (75.90%)	213 (77.70%)	0.256	0.613
	**No**	66 (24.10%)	61 (22.30%)		
	**Yes**				
**Onestage anastomosis**		66 (24.10%)	63 (23.00%)	0.091	0.763
	**No**	208 (75.90%)	211 (77.00%)		
	**Yes**				
**Perineum tamponade hemostatic**		262 (95.60%)	267 (97.40%)	1.363	0.243
	**No**	12 (4.40%)	7 (2.60%)		
	**Yes**				
**Postoperative complications**					
**Obstruction**		265 (96.70%)	270 (98.50%)	1.970	0.160
	**No**	9 (3.30%)	4 (1.50%)		
	**Yes**				
**Anastomotic fistula**		255 (93.10%)	264 (96.40%)	2.949	0.086
	**No**	19 (6.90%)	10 (3.60%)		
	**Yes**				
**Operative area infection**		234 (85.40%)	241 (88.00%)	0.774	0.379
	**No**	40 (14.60%)	33 (12.00%)		
	**Yes**				
**Cardiovascular disease**		271 (98.90%)	272 (99.30%)	0.000	1.000
	**No**	3 (1.10%)	2 (0.70%)		
	**Yes**				
**Length of stay (days)**		14 (11–18)	13 (10–15)	-4.384	**< 0.001**

P values considered statistically significant are presented in bold

**Fig. 2:**
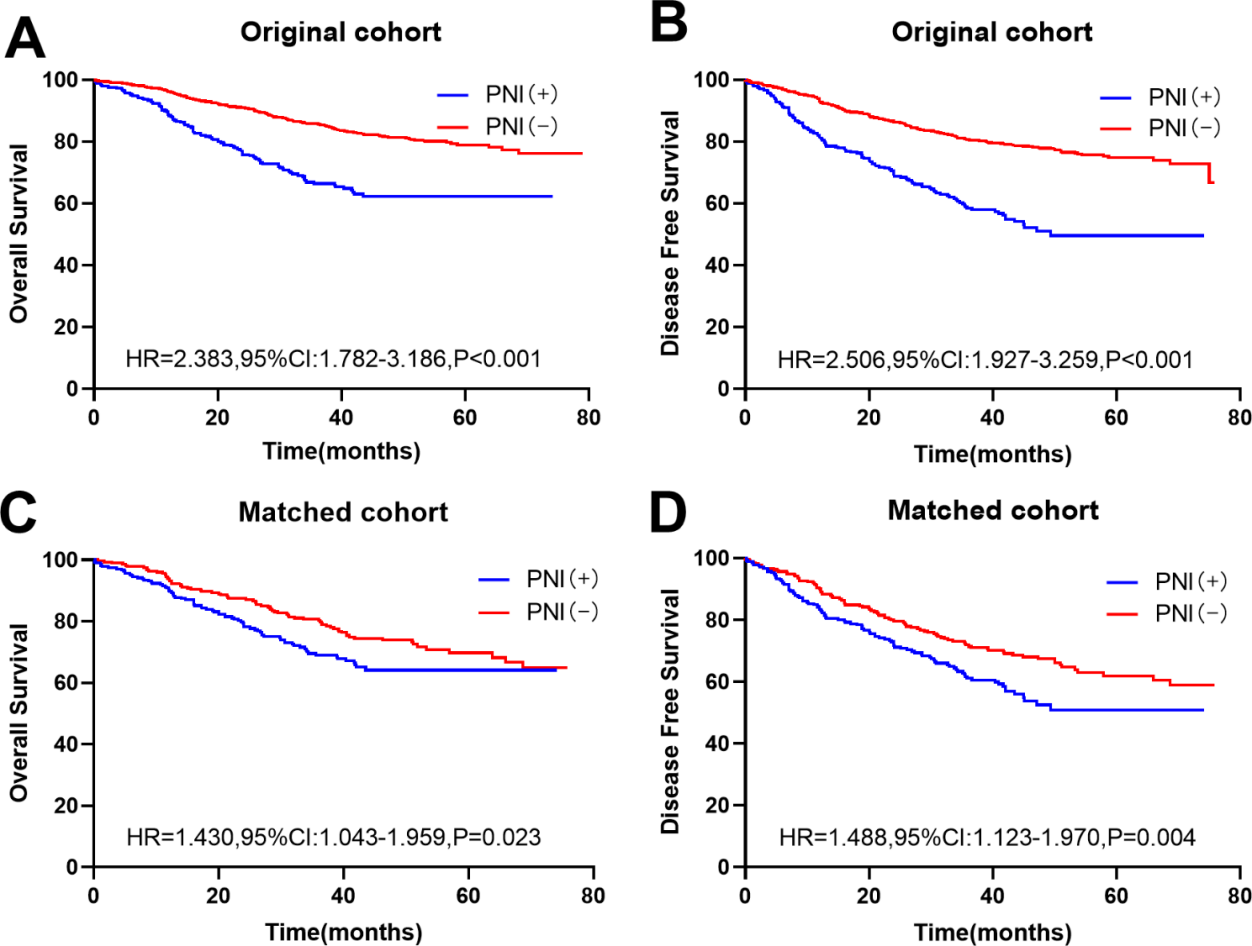
**Kaplan-Meier survival curves of patients grouped by perineural invasion (PNI).** The Kaplan-Meier survival curves of OS for patients grouped by PNI in the original cohort (A);The Kaplan-Meier survival curves of DFS for patients grouped by PNI in the original cohort (B);The Kaplan-Meier survival curves of OS for patients grouped by PNI in the matched cohort (C);The Kaplan-Meier survival curves of OS for patients grouped by PNI in the matched cohort (D)

The median follow-up time of the patients in this study was 42 (IQR, 0.167–79) months. The 5-year OS of patients in the PNI(+) group was significantly lower than that of patients in the PNI(-) group (68.10% vs. 82.50%, respectively), showing a statistically significant intergroup difference (HR, 2.383; 95% Cl, 1.782–3.186; P < 0.001) (Fig. 2A). The 5-year DFS of patients in the PNI(+) group was lower than that of patients in the PNI(-) group (59.60% vs. 78.50%), showing a statistically significant intergroup difference (HR, 2.506; 95% Cl, 1.927–3.259; P < 0.001) (Fig. 2B).

The median follow-up time after PSM was 40.4 (0.167–74.167) months. The 5-year OS of patients in the PNI(+) group was 69.7%, significantly lower than that in the PNI(-) group (73.0%) (HR, 1.430; 95% CI, 1.043–1.959; P = 0.023) (Fig. 2C). The 5-year DFS of patients in the PNI(+) group was 61.3%, significantly lower than that of patients in the PNI(-) group (66.40%) (HR, 1.488; 95% CI, 1.123–1.970; P = 0.004) (Fig. 2D).

**Fig. 3:**
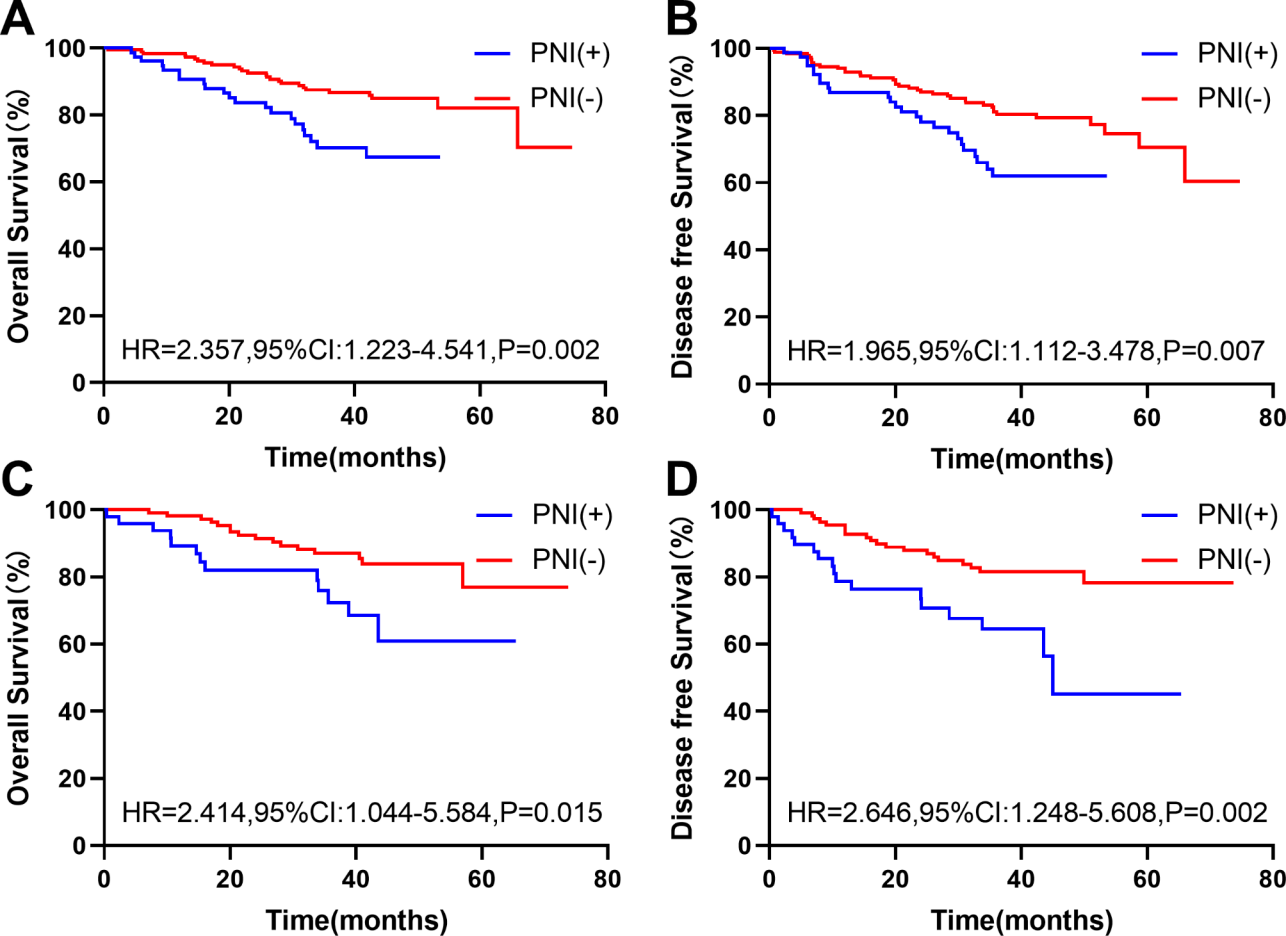
**The effect of PNI on OS and DFS of*KRAS*wild-type and mutant.** The Kaplan-Meier survival curves of the effect of PNI on OS(A) and DFS(B) of KRAS wild-type. The Kaplan-Meier survival curves of the effect of PNI on OS(C) and DFS(D) of KRAS mutant

In the original cohort, there were 419(28.6%) patients with a *KRAS* gene test result; of them, 261(62.29%) were wild-type and 158(37.71%) were mutant. In wild-type *KRAS* patients, compared with PNI(-) patients, PNI(+) patients had worse OS (HR, 2.357, 95% Cl: 1.223–4.541; P = 0.002) (Fig. 3A) and DFS (HR, 1.965, 95% CI: 1.112–3.478; P = 0.007) (Fig. 3B). The same results were obtained in *KRAS* mutated patients (OS: HR, 2.414, 95% CI: 1.044–5.584; P = 0.015; DFS: HR, 2.646, 95% CI: 1.248–5.608; P = 0.002) (Fig. 3C and D).

### Factors influencing patients’ OS and DFS

#### Uni- and multivariate analyses of OS

A Cox univariate analysis showed that age (≤ 60 years vs. > 60 years, P = 0.001), preoperative obstruction (P < 0.001), vascular tumour thrombus (P = 0.032), PNI (P = 0.024), postoperative chemotherapy (P < 0.001), degree of differentiation (poorly vs. moderately, P = 0.003), TNM stage (I vs. IV, P = 0.042), N stage (N0 vs. N1, N2, P < 0.001), M stage (P = 0.001), preoperative complications (P = 0.035), preoperative CEA (< 5 µg/L vs. ≥ 5 µg/L, P < 0.001), CA 19 − 9 (< 37 U/mL vs. ≥ 37 U/mL, P < 0.001), and CA125 level (< 35 U/mL vs. ≥ 35 U/mL, P < 0.001) were the prognostic factors of OS. The multivariate analysis showed preoperative intestinal obstruction (HR, 2.101; 95% CI, 1.393–3.168; P < 0.001), PNI (HR, 1.881; 95% CI, 1.350–2.620; P < 0.001), postoperative chemotherapy (HR, 0.497; 95% CI, 0.351–0.704; P < 0.001), N stage (N1: HR, 1.841; 95% CI, 1.222–2.775; P = 0.004; N2: HR, 2.418; 95% CI, 1.579–3.702; P < 0.001), M stage (HR, 2.311; 95% CI, 1.505–3.550; P < 0.001), preoperative CEA level (HR, 3.005; 95% CI, 1.960–4.608; P = 0.001), preoperative CA 19 − 9 level (HR, 1.597; 95% CI, 1.123–2.270; P = 0.009) are independent prognostic factors for OS (Table 3).

**Table 3:** Univariate and multivariate analyses of the prognostic factors for overall survival in the post-matching cohort

Variables		Univariate analysis		Multivariate analysis	
		HR (95%CI)	P	HR (95%CI)	P
Age (years)					
	**≤ 60**	Ref.	-	Ref.	-
	**> 60**	1.663 (1.215–2.276)	**0.001**	1.357 (0.953–1.932)	0.091
**BMI**		0.953 (0.903–1.005)	0.075		
**Tumor size (cm)**					
	**≤ 2.70**	Ref.	-		
	**2.70–4.40**	0.862 (0.519–1.432)	0.567		
	**> 4.40**	1.171 (0.716–1.914)	0.529		
**Obstruction before surgery**					
	**Absent**	Ref.	-	Ref.	-
	**present**	2.312 (1.62–3.299)	**< 0.001**	2.101 (1.393–3.168)	**< 0.001**
**Sex**					
	**Male**	Ref.	-		
	**Female**	0.965 (0.701–1.329)	0.826		
**Family history of cancer**					
	**No**	Ref.	-		
	**Yes**	0.793 (0.458–1.374)	0.408		
**Post radiotherapy**					
	**No**	Ref.	-		
	**Yes**	0.724 (0.339–1.546)	0.405		
**Adjuvant chemotherapy**					
	**No**	Ref.	-	Ref.	-
	**Yes**	0.537 (0.391–0.737)	**< 0.001**	0.497 (0.351–0.704)	**< 0.001**
**Vascular cancer embolus**					
	**Absent**	Ref.	-	Ref.	-
	**Present**	1.43 (1.032–1.982)	**0.032**	1.129 (0.791–1.611)	0.504
**Perineural invasion**					
	**Absent**	Ref.	-	Ref.	-
	**Present**	1.445 (1.051–1.986)	**0.024**	1.881 (1.35–2.62)	**< 0.001**
**Histological grade**					
	**Poorly**	Ref.	-	Ref.	-
	**Moderately**	0.568 (0.391–0.825)	**0.003**	0.69 (0.461–1.033)	0.071
	**Well**	0.703 (0.397–1.246)	0.228	1.034 (0.569–1.879)	0.914
**Stage**					
	**I**	Ref.	-		
	**II**	1.6 (0.215–11.92)	0.647		
	**III**	5.06 (0.705–36.295)	0.107		
	**IV**	7.861 (1.08-57.202)	**0.042**		
**T stage**					
	**T1**	Ref.	-		
	**T2**	0.152 (0.021–1.081)	0.060		
	**T3**	0.598 (0.147–2.429)	0.472		
	**T4**	0.884 (0.216–3.618)	0.864		
** N stage**					
	**N0**	Ref.	-	Ref.	-
	**N1**	2.08 (1.384–3.126)	**< 0.001**	1.841 (1.222–2.775)	**0.004**
	**N2**	2.661 (1.76–4.024)	**< 0.001**	2.311 (1.505–3.55)	**< 0.001**
**M stage**					
	**M0**	Ref.	-	Ref.	-
	**M1**	1.95 (1.333–2.852)	**0.001**	3.005 (1.96–4.608)	**< 0.001**
**Primary tumor location**					
	**Rectum**	Ref.	-		
	**Right colon**	1.236 (0.839–1.82)	0.284		
	**Left colon**	1.273 (0.875–1.853)	0.207		
**ASA**					
	**1**	Ref.	-		
	**2**	0.502 (0.159–1.584)	0.240		
	**3**	0.642 (0.197–2.091)	0.462		
	**4**	0.763 (0.217–2.679)	0.673		
**Previous history of abdominal surgery**					
	**No**	Ref.	-		
	**Yes**	1.227 (0.839–1.793)	0.292		
**Neoadjuvant chemotherapy**					
	**No**	Ref.	-		
	**Yes**	1.302 (0.663–2.554)	0.443		
**Preoperative comorbidities**					
**Total patients**					
	**No**	Ref.	-	Ref.	-
	**Yes**	1.424 (1.024–1.98)	**0.035**	1.319 (0.924–1.881)	0.127
**Cardiovascular disease**					
	**No**	Ref.	-		
	**Yes**	1.371 (0.97–1.938)	0.074		
**Cerebrovascular disease**					
	**No**	Ref.	-		
	**Yes**	1.447 (0.639–3.273)	0.375		
**COPD**					
	**No**	Ref.	-		
	**Yes**	1.227 (0.454–3.312)	0.687		
**Diabetes**					
	**No**	Ref.	-		
	**Yes**	1.362 (0.772–2.403)	0.286		
**CEA (ng/mL)**					
	**< 5**	Ref.	-	Ref.	-
	**≥ 5**	2.439 (1.759–3.384)	**< 0.001**	1.838 (1.291–2.617)	**0.001**
**CA199 (kU/L)**					
	**< 37**	Ref.	-	Ref.	-
	**≥ 37**	2.128 (1.537–2.948)	**< 0.001**	1.597 (1.123–2.27)	**0.009**
**CA125 (U/mL)**					
	**< 35**	Ref.	-	Ref.	-
	**≥ 35**	2.223 (1.485–3.33)	**< 0.001**	1.466 (0.932–2.305)	0.098

Abbreviations: BMI, body mass index (calculated as weight in kilograms divided by height in meters squared); ASA, American Society of Anesthesiologists Physical Status Classification; COPD, chronic obstructive pulmonary disease; CEA, carcino-embryonic antigen; CA19-9; CA12-5, carbohydrate antigen. P values considered statistically significant are presented in bold

#### Uni- and multivariate analyses of DFS

A univariate analysis of the Cox regression model showed that preoperative intestinal obstruction (P = 0.025), vascular tumour thrombus (P = 0.002), PNI (P = 0.005), degree of differentiation (poorly vs. moderately, P = 0.003), TNM stage (I vs. IV, P = 0.009), N stage (N0 vs. N1: P = 0.047; N0 vs. N2: P < 0.001), M stage (P < 0.001), preoperative CEA level (< 5 µg/L vs. ≥ 5 µg/L, P < 0.001), Preoperative CA 19 − 9 level (< 37 U/mL vs. ≥ 37 U/mL, P = 0.008), and preoperative CA125 level (< 35 U/mL vs. ≥ 35 U/mL, P = 0.030) were significant prognostic factors for DFS. A multivariate analysis showed that preoperative intestinal obstruction (HR, 1.734; 95% CI, 1.170–2.568; P = 0.006), PNI (HR, 1.800; 95% CI, 1.353–2.419; P < 0.001), moderate differentiation (HR, 0.689; 95% CI, 0.482–0.985; P = 0.041), N2 stage (HR, 1.976; 95% CI, 1.373–2.844; P < 0.001), M stage (HR, 3.270; 95% CI, 2.319–4.611; P < 0.001), and preoperative CEA level (HR, 1.52;95% CI, 1.120–2.063, P = 0.007) were independent prognostic factors of DFS (Table 4).

**Table 4:** Univariate and multivariate analyses of the prognostic factors for disease-free survival in the post-matching cohort

Variables		Univariate analysis		Multivariate analysis	
		**HR** (**95%CI**)	**P**	**HR** (**95%CI**)	**P**
**Age (years)**					
	**≤ 60**	Ref.	-		
	**> 60**	1.128 (0.851–1.494)	0.402		
**BMI**		0.99 (0.944–1.039)	0.691		
**Tumor size (cm)**					
	**≤ 2.70**	Ref.	-		
	**2.70–4.40**	0.954 (0.623–1.459)	0.827		
	**> 4.40**	0.854 (0.556–1.312)	0.473		
**Obstruction before surgery**					
	**Absent**	Ref.	-	Ref.	-
	**present**	1.494 (1.05–2.124)	**0.025**	1.734 (1.17–2.568)	**0.006**
**Sex**					
	**Male**	Ref.	-		
	**Female**	0.832 (0.627–1.104)	0.202		
**Family history of cancer**					
	**No**	Ref.	-		
	**Yes**	0.989 (0.629–1.555)	0.962		
**Post radiotherapy**					
	**No**	Ref.	-		
	**Yes**	0.735 (0.377–1.436)	0.368		
**Adjuvant chemotherapy**					
	**No**	Ref.	-		
	**Yes**	0.825 (0.624–1.091)	0.178		
**Vascular cancer embolus**					
	**Absent**	Ref.	-	Ref.	-
	**Present**	1.584 (1.189–2.109)	**0.002**	1.244 (0.913–1.696)	0.167
**Perineural invasion**					
	**Absent**	Ref.	-	Ref.	-
	**Present**	1.506 (1.134–2.001)	**0.005**	1.809 (1.353–2.419)	**< 0.001**
**Histological grade**					
	**Poorly**	Ref.	-	Ref.	-
	**Moderately**	0.601 (0.429–0.84)	**0.003**	0.689 (0.482–0.985)	**0.041**
	**Well**	0.66 (0.391–1.116)	0.121	0.749 (0.44–1.275)	0.287
**Stage**					
	**I**	Ref.	-		
	**II**	2.481 (0.339–18.173)	0.371		
	**III**	5.86 (0.818–41.989)	0.078		
	**IV**	14.046 (1.945-101.454)	**0.009**		
**T stage**					
	**T1**	Ref.	-		
	**T2**	0.247 (0.041–1.477)	0.125		
	**T3**	0.889 (0.22–3.596)	0.869		
	**T4**	1.145 (0.281–4.667)	0.850		
** N stage**					
	**N0**	Ref.	-	Ref.	-
	**N1**	1.434 (1.005–2.048)	**0.047**	1.314 (0.918–1.879)	0.135
	**N2**	2.397 (1.698–3.383)	**< 0.001**	1.976 (1.373–2.844)	**< 0.001**
**M stage**					
	**M0**	Ref.	-	Ref.	-
	**M1**	2.953 (2.145–4.066)	**< 0.001**	3.27 (2.319–4.611)	**< 0.001**
**Primary tumor location**					
	**Rectum**	Ref.	-		
	**Right colon**	0.9 (0.627–1.292)	0.567		
	**Left colon**	1.109 (0.796–1.544)	0.540		
**ASA**					
	**1**	Ref.	-		
	**2**	0.61 (0.194–1.918)	0.397		
	**3**	0.74 (0.229–2.389)	0.614		
	**4**	0.825 (0.239–2.85)	0.760		
**Previous history of abdominal surgery**					
	**No**	Ref.	-		
	**Yes**	0.842 (0.579–1.223)	0.366		
**Neoadjuvant chemotherapy**					
	**No**	Ref.	-		
	**Yes**	1.159 (0.613–2.192)	0.649		
**Preoperative comorbidities**					
**Total patients**					
	**No**	Ref.	-		
	**Yes**	1.238 (0.918–1.67)	0.161		
**Cardiovascular disease**					
	**No**	Ref.	-		
	**Yes**	1.227 (0.896–1.681)	0.203		
**Cerebrovascular disease**					
	**No**	Ref.	-		
	**Yes**	0.898 (0.37–2.184)	0.813		
**COPD**					
	**No**	Ref.	-		
	**Yes**	1.2 (0.493–2.916)	0.688		
**Diabetes**					
	**No**	Ref.	-		
	**Yes**	1.229 (0.726–2.082)	0.442		
**CEA (ng/mL)**					
	**< 5**	Ref.	-	Ref.	-
	**≥ 5**	1.839 (1.387–2.439)	**< 0.001**	1.52 (1.12–2.063)	**0.007**
**CA199 (kU/L)**					
	**< 37**	Ref.	-	Ref.	-
	**≥ 37**	1.517 (1.116–2.061)	**0.008**	1.147 (0.825–1.596)	0.414
**CA125 (U/mL)**					
	**< 35**	Ref.	-	Ref.	-
	**≥ 35**	1.558 (1.044–2.326)	**0.030**	1.193 (0.776–1.834)	0.420

**Abbreviations: BMI, body mass index (calculated as weight in kilograms divided by height in meters squared); ASA, American Society of Anesthesiologists Physical Status Classification; COPD, chronic obstructive pulmonary disease; CEA, carcino-embryonic antigen; CA19-9; CA12-5, carbohydrate antigen.**

**P values considered statistically significant are presented in bold.**

### Subgroup analysis of long-term prognostic factors in PNI(+) patients

The results of the Cox univariate analysis for PNI(+) patients showed that preoperative intestinal obstruction, postoperative chemotherapy, vascular tumour thrombus, degree of differentiation, N stage, M stage, preoperative combined with cerebrovascular disease, preoperative CEA level, preoperative CA 19 − 9 level, and preoperative CA125 level were prognostic factors for OS. The multivariate analysis showed that preoperative obstruction (HR, 2.718; 95% CI, 1.551–4.764; P < 0.001), vascular tumour thrombus (HR, 1.570; 95% CI, 1.001–2.462; P = 0.049), N stage (HR, 2.066; 95% CI, 1.166–3.663; P = 0.013; HR., 2.888; 95% CI, 1.558–5.353; P < 0.001), M stage (HR, 4.495; 95% CI, 2.380–8.492; P < 0.001), preoperative cerebral vascular disease (HR, 7.724; 95% CI, 2.624–22.738; P < 0.001), preoperative CEA level (HR, 1.884; 95% CI, 1.137–3.121; P = 0.014), preoperative CA 19 − 9 level (HR, 2.614; 95% CI, 1.607–4.250; P < 0.001), postoperative chemotherapy (HR, 0.346; 95% CI, 0.210–0.571; P < 0.001) is an independent prognostic factor of OS in PNI(+) patients (Supplementary Table 3).

Vascular invasion; intermediate differentiation; stage IV disease; N2 and M stages; and CEA, CA 19 − 9, and CA125 levels were prognostic factors of DFS. The multivariate analysis showed that vascular invasion (HR, 1.754; 95% CI, 1.188–2.592; P = 0.005), M stage (HR, 3.662; 95% CI, 2.314–5.795; P < 0.001), CEA (HR, 1.75; 95% CI, 1.152–2.659; P = 0.009), and CA 19 − 9 level (HR, 1.627; 95% CI, 1.062–2.493; P = 0.025) were independent prognostic factors of DFS (Supplementary Table 3). However, there was no significant difference in OS and DFS among patients with different disease stages (P > 0.05; Supplementary Fig. 1).

### Subgroup analysis of factors associated with chemotherapy

We conducted a subgroup analysis of the original cohort to discuss the impact of chemotherapy on different subgroups (Supplementary Tables 5 and 6). Our results showed that, compared with PNI(-) patients, a significantly higher proportion of PNI(+) patients were treated with postoperative chemotherapy (18.8% vs. 25.9%, P = 0.001). In addition, younger age, no preoperative intestinal obstruction, postoperative chemotherapy, presence of venous invasion, higher tumour stage, primary tumour in the rectum, lower ASA score, preoperative neoadjuvant chemotherapy, preoperative comorbidities, preoperative cardiovascular disease, lower preoperative CA125 level, and absence of postoperative intestinal obstruction and surgical site infection were all associated with a significantly increased proportion of postoperative chemotherapy use (P > 0.05). Univariate logistic regression analysis showed that PNI status was significantly related to whether patients chose chemotherapy (HR, 1.554, 95% CI: 1.247–1.937; P < 0.001). Furthermore, we also created a forest plot based on significant variables from the multivariate logistic regression analysis (Supplementary Fig. 2, Supplementary Table 7).

In both PNI(-) and PNI(+) groups, patients who received postoperative adjuvant chemotherapy had better OS than those who did not receive it(Original cohort: HR = 0.632, 95% C1: 0.479–0.834, P < 0.001; HR = 0.542, 95% C1: 0.364–0.807, P = 0.001; Matched cohort: HR = 0.600, 95% C1: 0.380–0.948, P = 0.028; HR = 0.464, 95% C1: 0.300-0.725, P < 0.001, Supplementary Fig. 3). In addition, Our results also showed that the OS of PNI(+) patients who received adjuvant chemotherapy was similar to that of PNI(-) patients who did not receive it in the PSM cohort(P > 0.05). The results demonstrated that postoperative chemotherapy significantly improved OS in PNI (+) patients.

### Subgroup analysis of effect of PNI on prognosis by primary tumour site

**Fig. 4:**
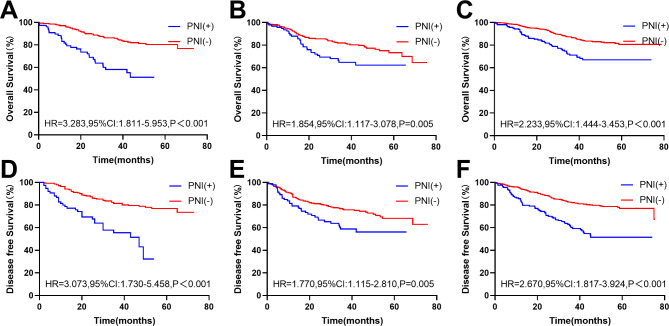
**Kaplan-Meier survival curves of patients with different tumor sites in the original cohort.** The Kaplan-Meier survival curves of the effect of PNI status on OS and DFS in the right colon(A/D), left colon(B/E), and rectum(C/F) in the original cohort

**Fig. 5:**
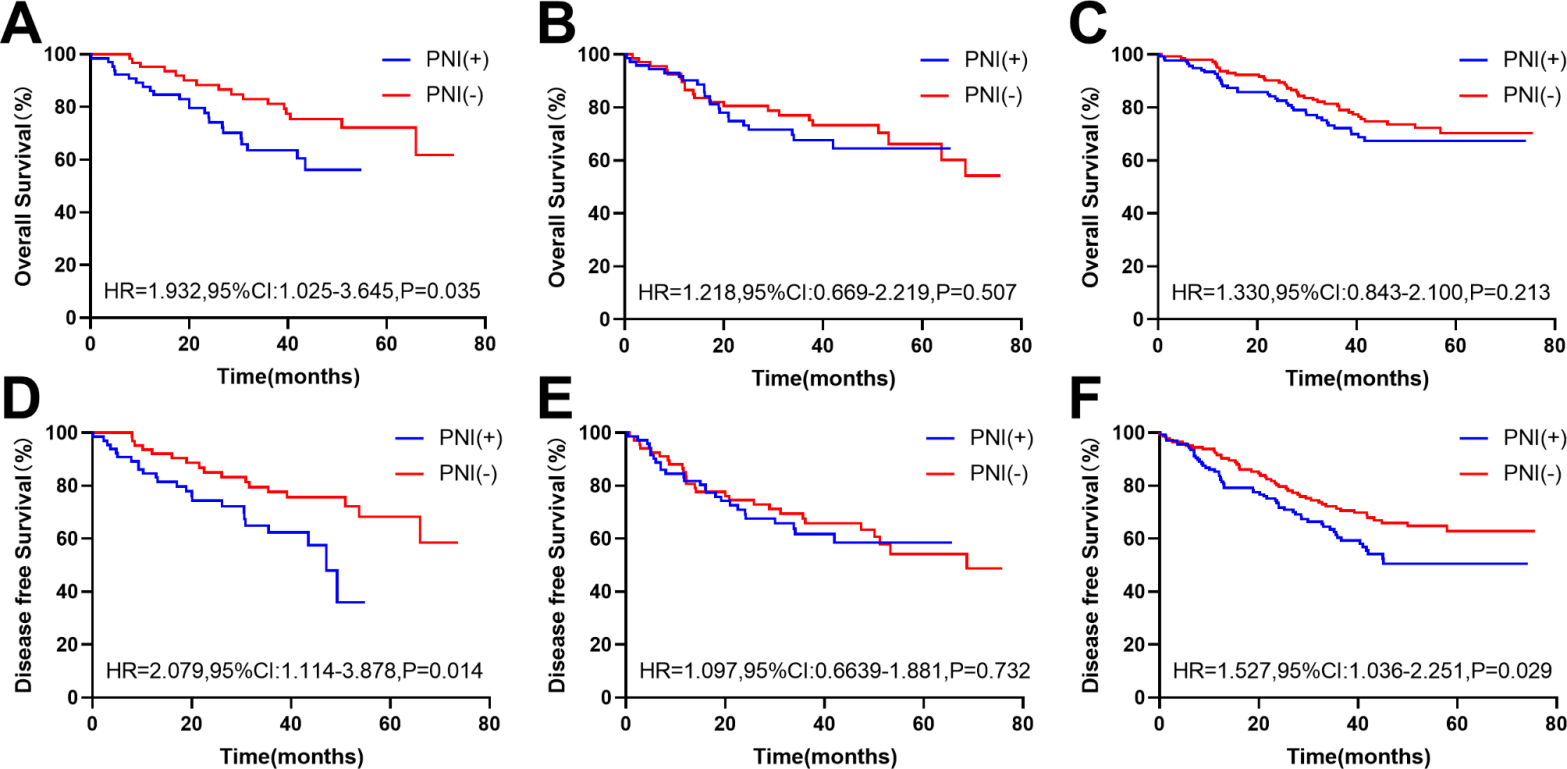
**Kaplan-Meier survival curves of patients with different tumor sites in the matched cohort.** The Kaplan-Meier survival curves of the effect of PNI status on OS and DFS in the right colon(A/D), left colon(B/E), and rectum(C/F) in the matched cohort

In the original cohort, compared with PNI(-) patients, PNI(+) patients had worse OS (HR, 3.283, 95% CI: 1.811–5.953; P < 0.001) and DFS (HR, 3.073, 95% CI: 1.730–5.458; P < 0.001) in the right colon (Fig. 4A and D). In addition, the same results were obtained in the left colon (OS: HR = 1.854, 95% CI: 1.117–3.078, P = 0.005; DFS: HR, 1.770, 95% CI: 1.115–2.810; P = 0.005) (Fig. 4B and E) and rectum (OS: HR, 2.233, 95% CI: 1.444–3.453; P < 0.001; DFS: HR, 2.670, 95%Cl: 1.817–3.924; P < 0.001) (Fig. 4C and F). In the matched cohort, compared with PNI(-) patients, PNI(+) patients had even worse OS (HR, 1.932, 95% CI: 1.025–3.645; P = 0.035) and DFS (HR, 2.079, 95% CI: 1.114–3.878; P = 0.014) in the right colon (Fig. 5A and D). There was no statistically significant difference in prognosis between the two groups of patients in the left colon and rectum (P > 0.05)(Fig. 5).

### Prediction model analysis of long-term patient prognosis

**Fig. 6:**
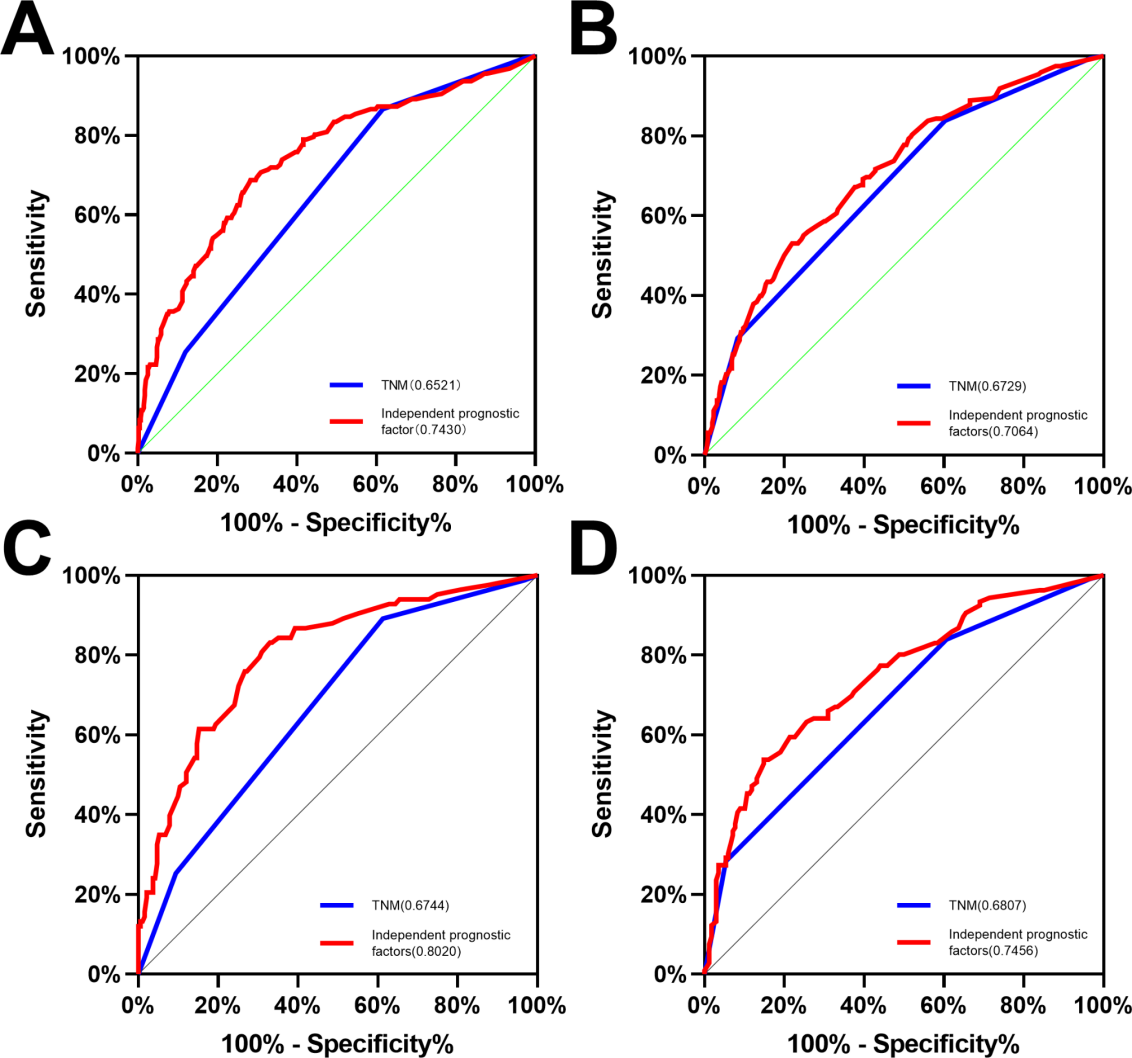
**Comparison of the receiver operating characteristic (ROC) curve of independent prognostic factors and TNM stage in patients.** Comparison of the Area under the curve(AUC) of OS(A) and DFS(B) of independent prognostic factors and TNM stage in patients grouped by PNI in the matched cohort; Comparison of AUC of OS(C) and DFS(D) of independent prognostic factors and TNM stage in PNI(+) subgroup after matching

Indicators of significance in our multifactorial analysis of Cox based on patient OS included preoperative bowel obstruction, PNI, postoperative chemotherapy, N stage, M stage, and preoperative CEA and CA 19 − 9 levels to construct a predictive model for OS. The AUROC is superior to the traditional TNM staging (0.743, 95% CI: 0.696–0.791 vs. 0.652, 95% CI: 0.603–0.701;P < 0.001) (Fig. 6A). Significant indicators in the Cox multifactorial analysis based on patient DFS included preoperative bowel obstruction, PNI, intermediate differentiation, N2 stage, M stage, and preoperative CEA level to construct a predictive model for DFS. Compared with the traditional TNM staging, our prediction model has greater advantages in predicting the prognosis of patients (0.706, 95% CI: 0.661–0.751 vs. 0.673, 95% CI: 0.622–0.720; P < 0.001) (Fig. 6B).

In the subgroup analysis of PNI(+) patients, we built a prediction model of OS based on seven indicators: preoperative intestinal obstruction, N stage, M stage, preoperative cerebral vascular disease, preoperative CEA and CA 19 − 9 levels, and postoperative chemotherapy use. The AUC was 0.802 vs. 0.674 (95%CI: 0.745–0.859 vs. 95% CI: 0.607–0.741; P < 0.001) (Fig. 6C). Based on vascular invasion, intermediate differentiation, N2 stage, M stage, and preoperative CEA and CA 19 − 9 levels, a predictive model of DFS was constructed with AUC of 0.746 vs. 0.681 (95% CI: 0.686–0.806 vs. 95% CI: 0.616–0.746; P < 0.001) (Fig. 6D).

## Discussion

This large single-centre retrospective clinical study focused on a special group of PNI(+) patients with CRC. We used PSM to compare the clinical, pathological, laboratory, and other indicators of PNI(+) and PNI(-) patients and explore the factors that affect the prognosis of patients in this particular disease state. Our results showed that the long-term prognosis of CRC patients with PNI(+) was poor and may be affected by different clinicopathological characteristics and perioperative results.

The main treatment for CRC is surgery, while adjuvant therapy, such as postoperative chemotherapy and radiotherapy, is also common. Although TNM staging is an important factor in determining the postoperative treatment mode and prognosis, TNM staging is less accurate in the early disease stages [13]. Therefore, relevant risk factors must be considered to identify patients who are likely to benefit from adjuvant therapy. Accurately judging the long-term prognosis of patients with CRC is of great significance for the clinical selection of treatment methods. Many indicators affect the prognosis of patients with CRC, but the conclusions of recent studies and the literature differ, among which the use of PNI as a prognostic indicator for patients with CRC after surgery is controversial. In this study, PSM was used to minimise the influence of each covariate on the study, explore its role in CRC, help predict patient prognosis, and determine further treatment options.

N and M staging are the gold standards for evaluating lymphatic and distant metastases. Patients with high disease stage are at higher risk of tumour invasion of the surrounding tissues, a higher risk of peripheral microvessel and lymphatic vessel invasion, a greater possibility of metastasis to distant organs, and an increased possibility of recurrence, resulting in poorer prognosis. Previous studies [14, 15] reported that T and N stages or TNM stages I–III can significantly affect the prognosis of CRC patients. Huh et al. [16] and Cao et al. [14] reported that preoperative CEA level, tumour size, TNM stage, and T and N stage were independent risk factors for a poor prognosis among PNI(+) patients. Al-Sukhni et al. [17] reported that lymphovascular and nerve invasion were associated with prognosis in advanced CRC, PNI was an independent poor prognostic marker for survival in CRC, and lymphovascular invasion and PNI were associated with lymph node involvement in T1 and T2 tumours. Zhou et al. [18] also found that tumour invasion depth and lymph node metastasis were independent prognostic factors affecting the survival of PNI(+) patients with CRC. The present study found that N and M stages were independent prognostic factors affecting OS and DFS in patients with PNI(+) CRC. The similarity of the results suggests that lymph node and distant metastasis are fully capable of influencing the prognosis of PNI(+) patients with CRC. Moreover, we used a more scientific PSM method to obtain more reliable results.

Previous studies reported a significant impact of PNI on the survival of surgical CRC patients with stage III or IV disease [19, 20]. Additionally, some studies suggested that PNI adversely affects the prognosis of surgical patients with stage I or II CRC. It is recommended that PNI be incorporated into TNM staging to screen high-risk patients and active intervention be provided to improve patient outcomes [16, 21, 22]. Overall, PNI is an unfavourable factor for prognosis at any disease stage, but its degree of impact varies. Here we compared the prognosis of PNI(+) and PNI(-) patients with stage I, II, III, and IV TNM classification. We found that PNI(+) patients with stage II disease had significantly worse DFS than PNI(-) patients with stage II disease (HR, 1.840, 95% CI: 1.004–3.370; P = 0.014); PNI positivity was also an adverse prognostic factor for OS and DFS in stage III and IV patients (III: HR, 1.732, 95% CI: 1.215–2.469; P < 0.001; IV: HR, 2.250, 95% CI: 1.337–3.786; P < 0.001) and DFS (III: HR = 1.742, 95% CI: 1.247–2.433; P < 0.001; IV: HR, 2.130, 95% CI: 1.368–3.315; P < 0.001) (Supplementary Fig. 4). Our results after PSM were not ideal, possibly because of a significantly reduced sample size and a large difference from the original sample size after screening. The analysis of a smaller number of patients within each stage was another contributing factor. However, our original cohort analysis results were similar to those of previous studies, supporting the impact of PNI on patients at different disease stages.

Preoperative intestinal obstruction in patients with CRC is a highly fatal acute disease with an incidence of approximately 8–29%. The prognosis of patients with CRC and preoperative intestinal obstruction is worse than that of CRC patients without intestinal obstruction [17, 23]. Due to the differences in study design, including different stages, curability, obstruction definition and degree, treatment strategies, and adjuvant chemotherapy, controversy persists on the research of prognostic factors of patients with obstructive CRC domestically and abroad. This study included a large patient population, of whom 13.5% had preoperative intestinal obstruction. The proportion of preoperative intestinal obstruction in PNI(+) patients before PSM was significantly higher than that in PNI(-) patients (21.70% vs. 11.10%, P < 0.001). Although there was no significant intergroup difference in the proportion of preoperative intestinal obstruction after PSM, more patients were affected in the PNI(+) than PNI(-) group (17.50% vs. 16.40%, P > 0.05). In contrast, the results of this study support that preoperative intestinal obstruction is an independent prognostic factor for OS in PNI(+) patients.

It is well known that vascular tumour emboli are closely related to tumour invasion and metastasis, and patients with vascular tumour emboli usually have comorbid lymph node metastasis. The American Joint Committee on Cancer (AJCC) and National Comprehensive Cancer Network (NCCN) clearly indicated that vascular invasion is one factor of poor prognosis in CRC [24, 25]. PNI can be an effective supplement to N and M staging [7, 9, 16] and be used to screen patients with advanced CRC to identify who are at high risk of a poor prognosis and assist clinical treatment to improve their survival rate. Sun et al. [15] reported that PNI, vascular tumour thrombi, TNM stage, and lymph node metastasis were independent factors affecting OS and DFS in patients with stage III CRC. Xu et al. [26] and Kim et al. [22] also reported that lymphovascular invasion, PNI, and poor differentiation were risk factors for a poor prognosis in cases of stage I CRC. Cao et al. [14] pointed out that lymphatic metastasis and vascular invasion are the main factors influencing PNI. Our results showed that among 332 PNI(+) patients, 45.70% had vascular tumour thrombi, 66.50% had lymph node metastasis, and 94.50% had stage T3–T4 disease, which may be one reason for the poor prognosis of PNI(+) patients. Moreover, our statistical results also suggested that lymphovascular invasion was a risk factor for OS and DFS in CRC patients and an independent prognostic factor for poor DFS in PNI(+) CRC patients.

Preoperative tumour marker detection is widely used in clinical practice because of its convenience and acceptability [27]. Serum CEA is a reliable tumour marker in CRC and recommended by the NCCN guidelines as a prognostic and monitoring indicator [24]. Preoperative CEA levels affect the prognosis of gastric cancer, lung cancer, CRC, and other tumours. CEA levels are also associated with metastasis and CRC recurrence after radical surgery [28–30]. In digestive system tumours, CA19-9 levels can be elevated; for CRC, elevated preoperative CA19-9 levels predict poor survival. Postoperative CA19-9 is a valuable prognostic indicator of lung and liver metastases [31]. It is worth noting that CA125 is not directly related to CRC, but it still garners the interest of prognostic analysis researchers. Huang et al. [32] reported that CA125 was associated with poor prognosis of CRC. Our study found that patients with elevated preoperative CEA and CA 19 − 9 levels had shorter long-term survival times and higher recurrence rates. Preoperative CEA was an independent prognostic factor for OS and DFS, while preoperative CEA and CA 19 − 9 levels were independent prognostic factors for OS and DFS in PNI(+) patients. In our study, preoperative CA125 level affected recurrence and patient survival and was a prognostic factor for recurrence and survival of PNI(+) patients, consistent with previous studies [31].

In recent years, with the increasing application of neoadjuvant and postoperative chemotherapy and the continuous improvement of chemotherapy regimens, the OS and DFS of patients with CRC have increased. To the best of our knowledge, an adjuvant treatment plan requires formulation by clinicians according to the individual differences among patients and indicators. Many studies suggested that CRC surgery and systemic chemotherapy can prolong patient survival time and that elderly [33], early-stage [28], or advanced [34] patients can benefit from postoperative adjuvant therapy. However, for CRC patients with PNI, no significant intergroup difference in survival time was noted. Therefore, whether patients benefit from postoperative chemotherapy remains controversial. Some studies [8, 10] suggested that traditional adjuvant chemotherapy could not improve the prognosis of PNI(+) CRC patients, while other studies [9, 22, 35] suggested that aggressive postoperative chemotherapy might have a protective effect on them. Moreover, according to the 7th edition of the AJCC manual [36], PNI is considered a site-specific prognostic indicator for CRC. The NCCN Clinical Practice guidelines [37] and the American Society of Clinical Oncology [38] also include PNI as a high-risk feature of CRC recurrence and recommend adjuvant therapy for patients with stage II CRC and PNI. The data from this study showed a 60.8% rate of postoperative chemotherapy in patients with PNI(+) CRC. The statistical analysis revealed that postoperative chemotherapy could significantly prolong the OS of patients with CRC and PNI(+) CRC but had no significant effect on DFS.

The traditional TNM stage or single index is often not sufficiently accurate to predict patient prognosis. The AUC of the OS multivariate model after PSM was 0.652, while that of the DFS multivariate model was 0.673. The receiver operating characteristic curve was constructed for the special groups. After PSM, the AUC of the OS and DFS multivariate models were 0.743 and 0.706, respectively. In the PNI(+) subgroup, the AUC of the 0 S variable model was 0.802, and the AUC of the DFS variable model was 0.746. Compared with traditional TNM staging, our prediction model has greater advantages in predicting the prognosis of patients. It is reasonable to believe that these independent prognostic factors combined with TNM staging can more accurately predict the prognosis of patients with CRC after surgery. The AUC of the model established in the PNI(+) subgroup was greater than 0.71, indicating that this model had good predictive ability. Compared with the multivariate model of ordinary patients, the independent predictors of PNI(+) patients could better predict the prognosis and survival of PNI(+) patients. This also indicates that the indicators used in the model can significantly affect the prognosis of patients with PNI(+) CRC.

This study aimed to investigate the impact of PNI on perioperative outcomes, recurrence, and long-term survival in patients undergoing CRC surgery. PSM can effectively avoid the possible bias caused by covariates, reduce the selection bias of research objects, and make the research results more convincing. In addition, the indicators included in this study are comprehensive, including clinical, pathological, postoperative complications, and long-term prognosis, which can more effectively predict the long-term prognosis of patients with CRC surgery and PNI(+) CRC surgery, and guide clinical treatment measures. However, this study has certain limitations. First, because this is a retrospective study, there will inevitably be some recall bias; prospective studies can be conducted to avoid such bias. Second, we used a single-centre database with a relatively limited number of patients, which may have affected the statistical results and reduced the value of our findings. A multicentre database was used to confirm the conclusions of this study. Finally, only the patients’ clinicopathological characteristics were included in this study. In the future, it will be necessary to further classify patients and include molecular or genetic indicators such as microsatellite phenotypes. Despite these limitations, our study demonstrates meaningful results that establish PNI(+) as an independent prognostic factor for OS and DFS in patients with CRC.

## Conclusion

In summary, our study demonstrated that the prognosis of patients with CRC after surgery was closely related to patient age, tumour differentiation, TNM stage, N stage, M stage, nerve invasion, vascular tumour thrombi, postoperative chemotherapy, preoperative obstruction, preoperative CEA level, preoperative CA 19 − 9 level, and other factors. PNI(+) is an independent risk factor for OS and DFS in patients with surgically treated CRC. In addition, postoperative chemotherapy can significantly improve the OS of PNI(+) patients; thus, it is recommended that all PNI(+) patients receive regular chemotherapy after surgery.

## Electronic supplementary material

Below is the link to the electronic supplementary material.

Supplementary Material 1

Supplementary Material 2

Supplementary Material 3

Supplementary Material 4

Supplementary Material 5

Supplementary Material 6

Supplementary Material 7

Supplementary Material 8

## Data Availability

The datasets generated and analyzed during the current study are available from the corresponding author upon reasonable request.

## Abbreviations

PNI: Perineural invasion
CRC: Colorectal cancer
PSM: Propensity score matching
OS: Overall survival
DFS: Disease-free survival
AUROC: Area under the receiver operating characteristic curves
HR: Hazard ratio
CI: Confidence interval
BMI: Body mass index
ASA: American Anaesthesiologist
CEA: Carcinoembryonic antigen
CA 19 − 9: Carbohydrate antigen 19 − 9
CA125: Cancer antigen 125
IQR: Interquartile range
AJCC: American Joint Committee on Cancer
NCCN: National Comprehensive Cancer Network
