# Supplementary material for: Perineural invasion affects prognosis of patients undergoing colorectal cancer surgery: a propensity score matching analysis
Source: BMC Cancer. 2023 May 18;23:452. doi: 10.1186/s12885-023-10936-w (PMC10197328; doi:10.1186/s12885-023-10936-w)
Supplement: Supplementary file 1 — Supplementary Material 1 [file 12885_2023_10936_MOESM1_ESM.docx]

**Supplementary Table 1** **Characteristics of patients in the original cohort.**

| **Characteristics** | | **PNI(-) (N=1138)** | **PNI(+) (N=332)** | **Z/X**2 | **P** |
| --- | --- | --- | --- | --- | --- |
| **Age (years)** | |  |  | 0.078 | 0.780 |
|  | **≤60** | 638 (56.10%) | 189 (56.90%) |  |  |
|  | **＞**60 | 500 (43.90%) | 143 (43.10%) |  |  |
| **BMI** | | 22.68 (20.90-24.49) | 22.63 (20.82-24.33) | -0.388 | 0.698 |
| **Tumor size (cm)** | |  |  | 29.678 | **<0.001** |
|  | **≤2.70** | 225 (19.80%) | 40 (12.00%) |  |  |
|  | **2.70-4.40** | 394 (34.60%) | 168 (50.60%) |  |  |
|  | **＞**4.40 | 519 (45.60%) | 124 (37.30%) |  |  |
| **Obstruction before surgery** | |  |  | 24.846 | **<0.001** |
|  | **Absent** | 1012 (88.9%) | 260 (78.3%) |  |  |
|  | **present** | 126 (11.1%) | 72 (21.7%) |  |  |
| **Sex** | |  |  | 1.354 | 0.245 |
|  | **Male** | 669 (58.8%) | 207 (62.3%) |  |  |
|  | **Female** | 469 (41.2%) | 125 (37.7%) |  |  |
| **Family history of cancer** | |  |  | 1.838 | 0.175 |
|  | **No** | 102 (9.00%) | 38 (11.4%) |  |  |
|  | **Yes** | 1036 (91.0%) | 294 (88.6%) |  |  |
| **Post radiotherapy** | |  |  | 2.219 | 0.136 |
|  | **No** | 1080 (94.9%) | 308 (92.8%) |  |  |
|  | **Yes** | 58 (5.10%) | 24 (7.20%) |  |  |
| **Adjuvant chemotherapy** | |  |  | 10.609 | **0.001** |
|  | **No** | 561 (49.3%) | 130 (39.2%) |  |  |
|  | **Yes** | 577 (50.7%) | 202 (60.8%) |  |  |
| **Vascular cancer embolus** | |  |  | 209.992 | **<0.001** |
|  | **Absent** | 1017 (89.4%) | 180 (54.2%) |  |  |
|  | **Present** | 121 (10.6%) | 152 (45.8%) |  |  |
| **Histological grade** | |  |  | -2.669 | **0.008** |
|  | **Poorly** | 162 (14.2%) | 54 (16.3%) |  |  |
|  | **Moderately** | 790 (69.4%) | 247 (74.4%) |  |  |
|  | **Well** | 186 (16.3%) | 31 (9.30%) |  |  |
| **Stage** | |  |  | -10.863 | **<0.001** |
|  | **Ⅰ** | 221 (19.4%) | 7 (2.10%) |  |  |
|  | **Ⅱ** | 447 (39.3%) | 82 (24.7%) |  |  |
|  | **Ⅲ** | 377 (33.1%) | 186 (56.0%) |  |  |
|  | **Ⅳ** | 93 (8.20%) | 57 (17.2%) |  |  |
| **T stage** | |  |  | -10.206 | **<0.001** |
|  | **T1** | 90 (7.90%) | 5 (1.50%) |  |  |
|  | **T2** | 210 (18.5%) | 13 (3.90%) |  |  |
|  | **T3** | 657 (57.7%) | 193 (58.1%) |  |  |
|  | **T4** | 181 (15.9%) | 121 (36.4%) |  |  |
| **N stage** | |  |  | -9.756 | **<0.001** |
|  | **N0** | 714 (62.7%) | 111 (33.4%) |  |  |
|  | **N1** | 272 (23.9%) | 123 (37.0%) |  |  |
|  | **N2** | 152 (13.4%) | 98 (29.5%) |  |  |
| **M stage** | |  |  | -4.150 | **<0.001** |
|  | **M0** | 1052 (92.4%) | 282 (84.9%) |  |  |
|  | **M1** | 86 (7.60%) | 50 (15.1%) |  |  |
| **Primary tumor location** | |  |  | 5.290 | 0.071 |
|  | **Right colon** | 282 (24.8%) | 77 (23.2%) |  |  |
|  | **Left colon** | 253 (22.2%) | 94 (28.3%) |  |  |
|  | **Rectum** | 603 (53.0%) | 161 (48.5%) |  |  |
| **ASA** | |  |  | 2.288 | 0.506 |
|  | **1** | 13 (1.10%) | 7 (2.10%) |  |  |
|  | **2** | 794 (69.80%) | 231 (69.60%) |  |  |
|  | **3** | 229 (20.10%) | 62 (18.70%) |  |  |
|  | **4** | 102 (9.00%) | 32 (9.60%) |  |  |
| **Previous history of abdominal surgery** | |  |  | 0.387 | 0.534 |
|  | **No** | 922 (81.00%) | 274 (82.50%) |  |  |
|  | **Yes** | 216 (19.00%) | 58 (17.50%) |  |  |
| **Neoadjuvant chemotherapy** | |  |  | 0.074 | 0.785 |
|  | **No** | 1073 (94.40%) | 312 (94.00%) |  |  |
|  | **Yes** | 64 (5.60%) | 20 (6.00%) |  |  |
| **preoperative comorbidities** | |  |  |  |  |
| **Total patients** | |  |  | 0.299 | 0.585 |
|  | **No** | 819 (72.00%) | 244 (73.50%) |  |  |
|  | **Yes** | 319 (28.00%) | 88 (26.50%) |  |  |
| **Cardiovascular disease** | |  |  | 1.183 | 0.277 |
|  | **No** | 869 (76.40%) | 263 (79.20%) |  |  |
|  | **Yes** | 269 (23.60%) | 69 (20.80%) |  |  |
| **Cerebrovascular disease** | |  |  | 0.963 | 0.326 |
|  | **No** | 1117 (98.20%) | 323 (97.30%) |  |  |
|  | **Yes** | 21 (1.80%) | 9 (2.70%) |  |  |
| **COPD** | |  |  | 0.000 | 0.990 |
|  | **No** | 1107 (97.30%) | 323 (97.30%) |  |  |
|  | **Yes** | 31 (2.70%) | 9 (2.70%) |  |  |
| **Diabetes** | |  |  | 1.550 | 0.213 |
|  | **No** | 1046 (91.90%) | 312 (94.00%) |  |  |
|  | **Yes** | 92 (8.10%) | 20 (6.00%) |  |  |
| **CEA (ng/mL)** | |  |  | 12.450 | **<0.001** |
|  | **<5** | 722 (63.40%) | 175 (52.70%) |  |  |
|  | **≥5** | 416 (36.60%) | 157 (47.30%) |  |  |
| **CA199 (kU/L)** | |  |  | 16.797 | **<0.001** |
|  | **<37** | 964 (84.70%) | 249 (75.00%) |  |  |
|  | **≥37** | 174 (15.30%) | 83 (25.00%) |  |  |
| **CA125 (U/mL)** | |  |  | 7.249 | **0.007** |
|  | **<35** | 1032 (90.70%) | 284 (85.50%) |  |  |
|  | **≥35** | 106 (9.30%) | 48 (14.50%) |  |  |
| **Abbreviations: BMI, body mass index (calculated as weight in kilograms divided by height in meters squared); ASA, American Society of Anesthesiologists Physical Status Classification; COPD, chronic obstructive pulmonary disease; CEA, carcino-embryonic antigen; CA19-9; CA12-5, carbohydrate antigen. P values considered statistically significant are presented in bold.** | | | | | |
