# Supplementary material for: Perineural invasion affects prognosis of patients undergoing colorectal cancer surgery: a propensity score matching analysis
Source: BMC Cancer. 2023 May 18;23:452. doi: 10.1186/s12885-023-10936-w (PMC10197328; doi:10.1186/s12885-023-10936-w)
Supplement: Supplementary file 2 — Supplementary Material 2 [file 12885_2023_10936_MOESM2_ESM.docx]

**Supplementary Table 2 Intraoperative management and postoperative complication outcomes in the original** **cohort.**

| **Characteristics** | | **PNI**(-) (N=1138) | **PNI**(+) (N=332) | **Z/X**2 | **P** |
| --- | --- | --- | --- | --- | --- |
| **Intraoperative management** | |  |  |  |  |
| **Type of surgery** | |  |  | 0.479 | 0.489 |
|  | **Laparoscopic** | 565 (49.60%) | 172 (51.80%) |  |  |
|  | **Laparotomy** | 573 (50.40%) | 160 (48.20%) |  |  |
| **Blood transfusion** | |  |  | 0.646 | 0.422 |
|  | **No** | 860 (75.60%) | 258 (77.70%) |  |  |
|  | **Yes** | 278 (24.40%) | 74 (22.30%) |  |  |
| **Onestage anastomosis** | |  |  | 3.029 | 0.082 |
|  | **No** | 221 (19.40%) | 79 (23.80%) |  |  |
|  | **Yes** | 917 (80.60%) | 253 (76.20%) |  |  |
| **Perineum tamponade hemostatic** | |  |  | 0.079 | 0.778 |
|  | **No** | 1111 (97.60%) | 325 (97.90%) |  |  |
|  | **Yes** | 27 (2.40%) | 7 (2.10%) |  |  |
| **Postoperative complications** | |  |  |  |  |
| **Obstruction** | |  |  | 1.067 | 0.302 |
|  | **No** | 1110 (97.50%) | 327 (98.50%) |  |  |
|  | **Yes** | 28 (2.50%) | 5 (1.50%) |  |  |
| **Anastomotic fistula** | |  |  | 0.730 | 0.393 |
|  | **No** | 1069 (93.90%) | 316 (95.20%) |  |  |
|  | **Yes** | 69 (6.10%) | 16 (4.80%) |  |  |
| **Operative area infection** | |  |  | 0.191 | 0.662 |
|  | **No** | 987 (86.70%) | 291 (87.70%) |  |  |
|  | **Yes** | 151 (13.30%) | 41 (12.30%) |  |  |
| **Cardiovascular disease** | |  |  | 0.021 | 0.884 |
|  | **No** | 1128 (99.10%) | 330 (99.40%) |  |  |
|  | **Yes** | 10 (0.90%) | 2 (0.60%) |  |  |
| **Length of stay (days)** | | 13 (11-17) | 13 (10-15) | -3.019 | **0.003** |
| **P values considered statistically significant are presented in bold.** | | | | | |
