# Supplementary material for: Perineural invasion affects prognosis of patients undergoing colorectal cancer surgery: a propensity score matching analysis
Source: BMC Cancer. 2023 May 18;23:452. doi: 10.1186/s12885-023-10936-w (PMC10197328; doi:10.1186/s12885-023-10936-w)
Supplement: Supplementary file 3 — Supplementary Material 3 [file 12885_2023_10936_MOESM3_ESM.docx]

**Supplementary table 3. Univariate and multivariate analyses of the prognostic factors for overall survival in the PNI group after PSM.**

| **Variables** | | | **Univariate analysis** | | **Multivariate analysis** | |
| --- | --- | --- | --- | --- | --- | --- |
|  |  |  | **HR** (95%CI) | **P** | **HR** (95%CI) | **P** |
| **Age (years)** | | |  |  |  |  |
|  | | **≤60** | Ref. | - |  |  |
|  | | **>**60 | 1.522 (0.989-2.342) | 0.056 |  |  |
| **BMI** | | | 0.967 (0.9-1.04) | 0.366 |  |  |
| **Tumor size (cm)** | | |  |  |  |  |
|  | | **≤2.70** | Ref. | - |  |  |
|  | | **2.70-4.40** | 0.814 (0.4-1.656) | 0.571 |  |  |
|  | | **>4.40** | 1.322 (0.662-2.64) | 0.429 |  |  |
| **Obstruction before surgery** | | |  |  |  |  |
|  | | **Absent** | Ref. | - | Ref. | - |
|  | | **present** | 2.356 (1.451-3.825) | **0.001** | 2.718 (1.551-4.764) | **<0.001** |
| **Sex** | | |  |  |  |  |
|  | | **Male** | Ref. | - |  |  |
|  | | **Female** | 1.204 (0.78-1.859) | 0.402 |  |  |
| **Family history of cancer** | | |  |  |  |  |
|  | | **No** | Ref. | - |  |  |
|  | | **Yes** | 0.53 (0.215-1.311) | 0.170 |  |  |
| **Post radiotherapy** | | |  |  |  |  |
|  | | **No** | Ref. | - |  |  |
|  | | **Yes** | 0.583 (0.213-1.592) | 0.292 |  |  |
| **Adjuvant chemotherapy** | | |  |  |  |  |
|  | | **No** | Ref. | - | Ref. | - |
|  | | **Yes** | 0.462 (0.3-0.713) | **<0.001** | 0.346 (0.21-0.571) | **<0.001** |
| **Vascular cancer embolus** | | |  |  |  |  |
|  | | **Absent** | Ref. | - | Ref. | - |
|  | | **Present** | 1.819 (1.18-2.802) | **0.007** | 1.482 (0.94-2.337) | **0.090** |
| **Histological grade** | | |  |  |  |  |
|  | | **Poorly** | Ref. | - | Ref. | - |
|  | | **Moderately** | 0.487 (0.286-0.83) | **0.008** | 0.663 (0.371-1.185) | 0.165 |
|  | | **Well** | 0.718 (0.322-1.599) | 0.417 | 1.189 (0.505-2.799) | 0.692 |
| **Stage** | | |  |  |  |  |
|  | | **Ⅰ** | Ref. | - |  |  |
|  | | **Ⅱ** | 0.803 (0.1-6.422) | 0.836 |  |  |
|  | | **Ⅲ** | 3.2 (0.442-23.151) | 0.249 |  |  |
|  | | **Ⅳ** | 6.476 (0.87-48.227) | 0.068 |  |  |
| **T stage** | | |  |  |  |  |
|  | | **T1** | Ref. | - |  |  |
|  | | **T2** | 0.137 (0.012-1.507) | 0.104 |  |  |
|  | | **T3** | 0.428 (0.103-1.773) | 0.242 |  |  |
|  | | **T4** | 1.27 (0.307-5.253) | 0.741 |  |  |
| **N stage** | | |  |  |  |  |
|  | | **N0** | Ref. | - | Ref. | - |
|  | | **N1** | 1.997 (1.139-3.502) | **0.016** | 2.066 (1.166-3.663) | **0.013** |
|  | | **N2** | 2.833 (1.594-5.036) | **<0.001** | 2.888 (1.558-5.353) | **0.001** |
| **M stage** | | |  |  |  |  |
|  | | **M0** | Ref. | - | Ref. | - |
|  | | **M1** | 2.524 (1.479-4.31) | **0.001** | 4.495 (2.38-8.492) | **<0.001** |
| **Primary tumor location** | | |  |  |  |  |
|  | | **Rectum** | Ref. | - |  |  |
|  | | **Right colon** | 1.404 (0.836-2.358) | 0.199 |  |  |
|  | | **Left colon** | 1.183 (0.699-2) | 0.532 |  |  |
| **ASA** | | |  |  |  |  |
|  | | **1** | Ref. | - |  |  |
|  | | **2** | 0.503 (0.157-1.161) | 0.248 |  |  |
|  | | **3** | 0.823 (0.245-2.277) | 0.753 |  |  |
|  | | **4** | 0.744 (0.197-2.280) | 0.663 |  |  |
| **Previous history of abdominal surgery** | | |  |  |  |  |
|  | | **No** | Ref. | - |  |  |
|  | | **Yes** | 1.237 (0.726-2.210) | 0.434 |  |  |
| **Neoadjuvant chemotherapy** | | |  |  |  |  |
|  | | **No** | Ref. | - |  |  |
|  | | **Yes** | 1.414 (0.652-3.307) | 0.381 |  |  |
| **Preoperative cardiovascular disease** | | |  |  |  |  |
|  | | **No** | Ref. | - |  |  |
|  | | **Yes** | 1.304 (0.788-2.215) | 0.301 |  |  |
| **Preoperative cerebrovascular disease** | | |  |  |  |  |
|  | | **No** | Ref. | - | Ref. | - |
|  | | **Yes** | 5.946 (2.169-16.163) | **0.001** | 7.724 (2.624-22.738) | **<0.001** |
| **Preoperative COPD** | | |  |  |  |  |
|  | | **No** | Ref. | - |  |  |
|  | | **Yes** | 1.052 (0.332-3.333) | 0.932 |  |  |
| **Preoperative diabetes** | | |  |  |  |  |
|  | | **No** | Ref. | - |  |  |
|  | | **Yes** | 1.045 (0.423-2.258) | 0.924 |  |  |
| **Any preoperative comorbidities** | | |  |  |  |  |
|  | | **No** | Ref. | - |  |  |
|  | | **Yes** | 1.542 (0.974-2.244) | 0.065 |  |  |
| **CEA (ng/mL)** | | |  |  |  |  |
|  | **<5** | | Ref. | - | Ref. | - |
|  | **≥5** | | 2.737 (1.727-4.433) | **<0.001** | 1.884 (1.137-3.121) | **0.014** |
| **CA199 (kU/L)** | | |  |  |  |  |
|  | **<37** | | Ref. | - | Ref. | - |
|  | **≥37** | | 2.742 (1.771-4.424) | **<0.001** | 2.614 (1.607-4.253) | **<0.001** |
| **CA125 (U/mL)** | | |  |  |  |  |
|  | **<35** | | Ref. | - | Ref. | - |
|  | **≥35** | | 2.732 (1.578-4.472) | **<0.001** | 1.38 (0.751-2.535) | 0.300 |
| **Abbreviations: BMI, body mass index (calculated as weight in kilograms divided by height in meters squared); ASA, American Society of Anesthesiologists Physical Status Classification; COPD, chronic obstructive pulmonary disease; CEA, carcino-embryonic antigen; CA19-9; CA12-5, carbohydrate antigen. Bold was used to highlight values that were statistically significant (<0.05) .** | | | | | | |
