# Supplementary material for: Perineural invasion affects prognosis of patients undergoing colorectal cancer surgery: a propensity score matching analysis
Source: BMC Cancer. 2023 May 18;23:452. doi: 10.1186/s12885-023-10936-w (PMC10197328; doi:10.1186/s12885-023-10936-w)
Supplement: Supplementary file 5 — Supplementary Material 5 [file 12885_2023_10936_MOESM5_ESM.docx]

**Supplementary table 5. Characteristics of patients grouped according to presence or absence of postoperative chemotherapy in the original cohort.**

| **Characteristics** | | **All (N=1470)** | **No (N=691)** | **Yes** (N=779) | **z/x2** | **P** |
| --- | --- | --- | --- | --- | --- | --- |
| **Age (years)** | |  |  |  | 87.402 | **＜**0.001 |
|  | **≤60** | 827 (56.30%) | 300 (43.40%) | 527 (67.70%) |  |  |
|  | **＞**60 | 643 (43.70%) | 391 (56.60%) | 252 (32.30%) |  |  |
| **BMI** | | 1470 | 22.86 (3.53) | 22.58 (3.66) | -0.030 | 0.976 |
| **Tumor size (cm)** | | | | | 4.832 | 0.089 |
|  | **≤2.70** | 265 (18.00%) | 123 (17.80%) | 142 (18.20%) |  |  |
|  | **2.70-4.40** | 562 (38.20%) | 246 (35.60%) | 316 (40.60%) |  |  |
|  | **＞**4.40 | 643 (43.70%) | 322 (46.60%) | 321 (41.20%) |  |  |
| **Obstruction before surgery** | | | | | 9.304 | **0.002** |
|  | **Absent** | 1272 (86.50%) | 578 (83.60%) | 694 (89.10%) |  |  |
|  | **present** | 198 (13.50%) | 113 (16.40%) | 85 (10.90%) |  |  |
| **Sex** | | | | | 0.056 | 0.813 |
|  | **Male** | 594 (40.40%) | 277 (40.10%) | 317 (40.70%) |  |  |
|  | **Female** | 876 (59.60%) | 414 (59.90%) | 462 (59.30%) |  |  |
| **Smoke** | | | | | 0.058 | 0.809 |
|  | **No** | 1134 (77.10%) | 535 (77.40%) | 599 (76.90%) |  |  |
|  | **Yes** | 336 (22.90%) | 156 (22.60%) | 180 (23.10%) |  |  |
| **Family history of cancer** | | | | | 3.703 | 0.054 |
|  | **No** | 1330 (90.50%) | 636 (92.00%) | 694 (89.10%) |  |  |
|  | **Yes** | 140 (9.50%) | 55 (8.00%) | 85 (10.90%) |  |  |
| **Post radiotherapy** | | | | | 65.510 | **＜**0.001 |
|  | **No** | 1388 (94.40%) | 688 (99.60%) | 700 (89.90%) |  |  |
|  | **Yes** | 82 (5.60%) | 3 (0.40%) | 79 (10.10%) |  |  |
| **Vascular cancer embolus** | | | | | 4.815 | **0.028** |
|  | **Absent** | 1197 (81.40%) | 579 (83.80%) | 618 (79.30%) |  |  |
|  | **Present** | 273 (18.60%) | 112 (16.20%) | 161 (20.70%) |  |  |
| **Peripheral nerve invasion** | | | | | 10.609 | **0.001** |
|  | **Absent** | 1138 (77.40%) | 561 (81.20%) | 577 (74.10%) |  |  |
|  | **Present** | 332 (22.60%) | 130 (18.80%) | 202 (25.90%) |  |  |
| **Histological grade** | | | | | 2.494 | 0.287 |
|  | **Poorly differentiated** | 216 (14.70%) | 112 (16.20%) | 104 (13.40%) |  |  |
|  | **Moderately differentiated** | 1037 (70.50%) | 481 (69.60%) | 556 (71.40%) |  |  |
|  | **Well differentiated** | 217 (14.80%) | 98 (14.20%) | 119 (15.30%) |  |  |
| **Stage** | |  |  |  | 33.374 | **＜**0.001 |
|  | **Ⅰ** | 228 (15.50%) | 130 (18.80%) | 98 (12.60%) |  |  |
|  | **Ⅱ** | 529 (36.00%) | 264 (38.20%) | 265 (34.00%) |  |  |
|  | **Ⅲ** | 563 (38.30%) | 255 (36.90%) | 308 (39.50%) |  |  |
|  | **Ⅳ** | 150 (10.20%) | 42 (6.10%) | 108 (13.90%) |  |  |
| **T stage** | |  |  |  | 12.524 | **0.006** |
|  | **T1** | 95 (6.50%) | 57 (8.20%) | 38 (4.90%) |  |  |
|  | **T2** | 223 (15.20%) | 119 (17.20%) | 104 (13.40%) |  |  |
|  | **T3** | 850 (57.80%) | 381 (55.10%) | 469 (60.20%) |  |  |
|  | **T4** | 302 (20.50%) | 134 (19.40%) | 168 (21.60%) |  |  |
| **N stage** | |  |  |  | 6.611 | **0.037** |
|  | **N0** | 825 (56.10%) | 412 (59.60%) | 413 (53.00%) |  |  |
|  | **N1** | 395 (26.90%) | 173 (25.00%) | 222 (28.50%) |  |  |
|  | **N2** | 250 (17.00%) | 106 (15.30%) | 144 (18.50%) |  |  |
| **M stage** | |  |  |  | 23.588 | **＜**0.001 |
|  | **M0** | 1334 (90.70%) | 654 (94.60%) | 680 (87.30%) |  |  |
|  | **M1** | 136 (9.30%) | 37 (5.40%) | 99 (12.70%) |  |  |
| **Primary tumor location** | | | | | 7.827 | **0.020** |
|  | **Right colon** | 359 (24.40%) | 186 (26.90%) | 173 (22.20%) |  |  |
|  | **Left colon** | 347 (23.60%) | 172 (24.90%) | 175 (22.50%) |  |  |
|  | **Rectum** | 764 (52.00%) | 333 (48.20%) | 431 (55.30%) |  |  |
| **ASA** | |  |  |  | 17.267 | **0.001** |
|  | **1** | 20 (1.40%) | 5 (0.70%) | 15 (1.90%) |  |  |
|  | **2** | 1025 (69.70%) | 458 (66.30%) | 567 (72.80%) |  |  |
|  | **3** | 291 (19.80%) | 147 (21.30%) | 144 (18.50%) |  |  |
|  | **4** | 134 (9.10%) | 81 (11.70%) | 53 (6.80%) |  |  |
| **Previous history of abdominal surgery** | | | | | 0.026 | 0.872 |
|  | **No** | 1196 (81.40%) | 561 (81.20%) | 635 (81.50%) |  |  |
|  | **Yes** | 274 (18.60%) | 130 (18.80%) | 144 (18.50%) |  |  |
| **Neoadjuvant chemotherapy** | | | | | 44.067 | **＜**0.001 |
|  | **No** | 1386 (94.30%) | 681 (98.60%) | 705 (90.50%) |  |  |
|  | **Yes** | 84 (5.70%) | 10 (1.40%) | 74 (9.50%) |  |  |
| **Preoperative comorbidities** | | | | |  |  |
| **Total patient** | |  |  |  | 10.452 | **0.001** |
|  | **No** | 1063 (72.30%) | 472 (68.30%) | 591 (75.90%) |  |  |
|  | **Yes** | 407 (27.70%) | 219 (31.70%) | 188 (24.10%) |  |  |
| **Cardiovascular disease** | | | | | 10.520 | **0.001** |
|  | **No** | 1132 (77.00%) | 506 (73.20%) | 626 (80.40%) |  |  |
|  | **Yes** | 338 (23.00%) | 185 (26.80%) | 153 (19.60%) |  |  |
| **Cerebrovascular disease** | | | | | 2.076 | 0.150 |
|  | **No** | 1440 (98.00%) | 673 (97.40%) | 767 (98.50%) |  |  |
|  | **Yes** | 30 (2.00%) | 18 (2.60%) | 12 (1.50%) |  |  |
| **COPD** | |  |  |  | 2.787 | 0.095 |
|  | **No** | 1430 (97.30%) | 667 (96.50%) | 763 (97.90%) |  |  |
|  | **Yes** | 40 (2.70%) | 24 (3.50%) | 16 (2.10%) |  |  |
| **Diabetes** | |  |  |  | 2.707 | 0.100 |
|  | **No** | 1358 (92.40%) | 630 (91.20%) | 728 (93.50%) |  |  |
|  | **Yes** | 112 (7.60%) | 61 (8.80%) | 51 (6.50%) |  |  |
| **CEA (ng/mL)** | |  |  |  | 0.153 | 0.696 |
|  | **<5** | 897 (61.00%) | 418 (60.50%) | 479 (61.50%) |  |  |
|  | **≥5** | 573 (39.00%) | 273 (39.50%) | 300 (38.50%) |  |  |
| **CA199 (kU/L)** | |  |  |  | 0.149 | 0.699 |
|  | **<37** | 1213 (82.50%) | 573 (82.90%) | 640 (82.20%) |  |  |
|  | **≥37** | 257 (17.50%) | 118 (17.10%) | 139 (17.80%) |  |  |
| **CA125 (U/mL)** | |  |  |  | 8.033 | **0.005** |
|  | **<35** | 1316 (89.50%) | 602 (87.10%) | 714 (91.70%) |  |  |
|  | **≥35** | 154 (10.50%) | 89 (12.90%) | 65 (8.30%) |  |  |
| **Abbreviations: BMI, body mass index (calculated as weight in kilograms divided by height in meters squared); ASA, American Society of Anesthesiologists Physical Status Classification; COPD, chronic obstructive pulmonary disease; CEA, carcino-embryonic antigen; CA19-9; CA12-5, carbohydrate antigen.** | | | | | | |
