# Supplementary material for: Perineural invasion affects prognosis of patients undergoing colorectal cancer surgery: a propensity score matching analysis
Source: BMC Cancer. 2023 May 18;23:452. doi: 10.1186/s12885-023-10936-w (PMC10197328; doi:10.1186/s12885-023-10936-w)
Supplement: Supplementary file 6 — Supplementary Material 6 [file 12885_2023_10936_MOESM6_ESM.docx]

**Supplementary table 6. Intraoperative management and postoperative complication outcomes in the original cohort.**

| **Characteristics** | | **All (N=1470)** | **No (N=691)** | **Yes** (N=779) | **z/x2** | **P Value** |
| --- | --- | --- | --- | --- | --- | --- |
| **Intraoperative management** | | | | |  |  |
| **Type of surgery** | |  |  |  | 1.430 | 0.232 |
|  | **Laparoscopic** | 737 (50.10%) | 335 (48.50%) | 402 (51.60%) |  |  |
|  | **Laparotomy** | 733 (49.90%) | 356 (51.50%) | 377 (48.40%) |  |  |
| **Blood transfusion** | |  |  |  | 1.996 | 0.158 |
|  | **No** | 1118 (76.10%) | 514 (74.40%) | 604 (77.50%) |  |  |
|  | **Yes** | 352 (23.90%) | 177 (25.60%) | 175 (22.50%) |  |  |
| **Primary anastomosis** | | | | | 2.413 | 0.120 |
|  | **No** | 300 (20.40%) | 153 (22.10%) | 147 (18.90%) |  |  |
|  | **Yes** | 1170 (79.60%) | 538 (77.90%) | 632 (81.10%) |  |  |
| **Perineum tamponade hemostatic** | | | | | 0.117 | 0.733 |
|  | **No** | 1436 (97.70%) | 676 (97.80%) | 760 (97.60%) |  |  |
|  | **Yes** | 34 (2.30%) | 15 (2.20%) | 19 (2.40%) |  |  |
| **Postoperative complications** | | | | |  |  |
| **Obstruction** | |  |  |  | 5.238 | **0.022** |
|  | **No** | 1437 (97.80%) | 669 (96.80%) | 768 (98.60%) |  |  |
|  | **Yes** | 33 (2.20%) | 22 (3.20%) | 11 (1.40%) |  |  |
| **Anastomotic fistula** | | | | | 2.487 | 0.115 |
|  | **No** | 1385 (94.20%) | 644 (93.20%) | 741 (95.10%) |  |  |
|  | **Yes** | 85 (5.80%) | 47 (6.80%) | 38 (4.90%) |  |  |
| **Operative area infection** | | | | | 7.574 | **0.006** |
|  | **No** | 1278 (86.90%) | 583 (84.40%) | 695 (89.20%) |  |  |
|  | **Yes** | 192 (13.10%) | 108 (15.60%) | 84 (10.80%) |  |  |
| **Cardiovascular disease** | | | | | 0.623 | 0.430 |
|  | **No** | 1458 (99.20%) | 684 (99.00%) | 774 (99.40%) |  |  |
|  | **Yes** | 12 (0.80%) | 7 (1.00%) | 5 (0.60%) |  |  |
| **Length of stay (days)** | | 1470 | 13 (5) | 13 (5) | -1.291 | 0.197 |
| **Bold was used to highlight values that were statistically significant (<0.05) .** | | | | | | |
