# Supplementary material for: Perineural invasion affects prognosis of patients undergoing colorectal cancer surgery: a propensity score matching analysis
Source: BMC Cancer. 2023 May 18;23:452. doi: 10.1186/s12885-023-10936-w (PMC10197328; doi:10.1186/s12885-023-10936-w)
Supplement: Supplementary file 7 — Supplementary Material 7 [file 12885_2023_10936_MOESM7_ESM.docx]

**Supplementary table 7. Univariate and multivariate [logistic regression](javascript:;) analyses of factors related to postoperative chemotherapy in the original cohort.**

| **Variables** | | **Univariate analysis** | **P** | **Multivariate analysis** | **P** |
| --- | --- | --- | --- | --- | --- |
|  |  | **HR** (95%CI) |  | **HR** (95%CI) |  |
| **Age (years)** | | | | |  |
|  | **≤60** |  |  |  |  |
|  | **＞**60 | 0.367 (0.297-0.454) | **＜**0.001 | 0.390 (0.306-0.497) | **＜**0.001 |
| **BMI** | | 1.005 (1.001-1.010) | **0.018** | 1.001 (0.969-1.034) | 0.970 |
| **Tumor size (cm)** | | | | |  |
|  | **≤2.70** |  |  |  |  |
|  | **2.70-4.40** | 1.285 (1.087-1.517) | **0.003** | 1.440 (1.001-2.071) | **0.049** |
|  | **＞**4.40 | 0.997 (0.854-1.164) | 0.969 | 1.186 (0.817-1.722) | 0.370 |
| **Obstruction before surgery** | | | | |  |
|  | **Absent** |  |  |  |  |
|  | **present** | 0.752 (0.568-0.997) | **0.047** | 0.720 (0.502-1.032) | 0.073 |
| **Sex** | | | | |  |
|  | **Male** |  |  |  |  |
|  | **Female** | 1.116 (0.977-1.274) | 0.105 |  |  |
| **Smoke** | | | | |  |
|  | **No** |  |  |  |  |
|  | **Yes** | 1.154 (0.931-1.430) | 0.191 |  |  |
| **Family history of cancer** | | | | |  |
|  | **No** |  |  |  |  |
|  | **Yes** | 1.545 (1.101-2.169) | **0.012** | 1.384 (0.942-2.035) | 0.098 |
| **Post radiotherapy** | | | | |  |
|  | **No** |  |  |  |  |
|  | **Yes** | 26.333 (8.314-83.405) | **＜**0.001 | 19.772 (6.007-65.080) | **＜**0.001 |
| **Vascular cancer embolus** | | | | |  |
|  | **Absent** |  |  |  |  |
|  | **Present** | 1.437 (1.129-1.830) | **0.003** | 1.143 (0.825-1.584) | 0.421 |
| **Peripheral nerve invasion** | | | | |  |
|  | **Absent** |  |  |  |  |
|  | **Present** | 1.554 (1.247-1.937) | **＜**0.001 | 1.220 (0.898-1.657) | 0.203 |
| **Histological grade** | | | | |  |
|  | **Poorly differentiated** |  |  |  |  |
|  | **Moderately differentiated** | 1.156 (1.023-1.306) | **0.020** | 1.308 (0.945-1.812) | 0.105 |
|  | **Well differentiated** | 1.214 (0.929-1.586) | 0.155 | 1.199 (0.789-1.824) | 0.395 |
| **Stage** | |  |  |  |  |
|  | **Ⅰ** |  |  |  |  |
|  | **Ⅱ** | 1.004 (0.846-1.190) | 0.965 | 1.079 (0.590-1.973) | 0.806 |
|  | **Ⅲ** | 1.208 (1.023-1.426) | **0.026** | 1.621 (0.674-3.900) | 0.281 |
|  | **Ⅳ** | 2.571 (1.800-3.673) | **＜**0.001 | 2.478 (0.591-10.399) | 0.215 |
| **T stage** | |  |  |  |  |
|  | **T1** |  |  |  |  |
|  | **T2** | 0.874 (0.672-1.137) | 0.316 | 1.420 (0.823-2.449) | 0.207 |
|  | **T3** | 1.231 (1.075-1.409) | **0.003** | 1.739 (0.872-3.466) | 0.116 |
|  | **T4** | 1.254 (0.999-1.573) | 0.051 | 1.877 (0.917-3.842) | 0.085 |
| **N stage** | |  |  |  |  |
|  | **N0** |  |  |  |  |
|  | **N1** | 1.283 (1.052-1.565) | **0.014** | 0.779 (0.379-1.599) | 0.495 |
|  | **N2** | 1.358 (1.057-1.746) | **0.017** | 0.740 (0.357-1.533) | 0.418 |
| **M stage** | |  |  |  |  |
|  | **M0** |  |  |  |  |
|  | **M1** | 2.676 (1.834-3.903) | **＜**0.001 | 0.954 (0.254-3.590) | 0.944 |
| **Primary tumor location** | | | | |  |
|  | **Right colon** |  |  |  |  |
|  | **Left colon** | 1.017 (0.824-1.256) | 0.872 | 1.057 (0.765-1.460) | 0.736 |
|  | **Rectum** | 1.294 (1.122-1.493) | **＜**0.001 | 1.290 (0.955-1.742) | 0.097 |
| **ASA** | |  |  |  |  |
|  | **1** |  |  |  |  |
|  | **2** | 1.238 (1.095-1.400) | **0.001** | 0.282 (0.123-0.645) | **0.003** |
|  | **3** | 0.980 (0.778-1.233) | 0.860 | 0.309 (0.129-0.740) | **0.008** |
|  | **4** | 0.654 (0.463-0.925) | **0.016** | 0.263 (0.106-0.652) | **0.004** |
| **Previous history of abdominal surgery** | | | | |  |
|  | **No** |  |  |  |  |
|  | **Yes** | 1.108 (0.874-1.404) | 0.398 |  |  |
| **Neoadjuvant chemotherapy** | | | | |  |
|  | **No** |  |  |  |  |
|  | **Yes** | 7.400 (3.823-14.322) | **＜**0.001 | 4.386 (2.125-9.053) | **＜**0.001 |
| **Preoperative comorbidities** | | | | |  |
| **Total patient** | |  |  |  |  |
|  | **No** |  |  |  |  |
|  | **Yes** | 0.858 (0.706-1.043) | 0.125 |  |  |
| **Cardiovascular disease** | | | | |  |
|  | **No** |  |  |  |  |
|  | **Yes** | 0.827 (0.668-1.025) | 0.082 |  |  |
| **Cerebrovascular disease** | | | | |  |
|  | **No** |  |  |  |  |
|  | **Yes** | 0.667 (0.321-1.384) | 0.277 |  |  |
| **COPD** | |  |  |  |  |
|  | **No** |  |  |  |  |
|  | **Yes** | 0.667 (0.354-1.255) | 0.209 |  |  |
| **Diabetes** | |  |  |  |  |
|  | **No** |  |  |  |  |
|  | **Yes** | 0.836 (0.576-1.213) | 0.345 |  |  |
| **CEA (ng/mL)** | |  |  |  |  |
|  | **<5** |  |  |  |  |
|  | **≥5** | 1.099 (0.933-1.295) | 0.260 |  |  |
| **CA199 (kU/L)** | |  |  |  |  |
|  | **<37** |  |  |  |  |
|  | **≥37** | 1.178 (0.922-1.506) | 0.191 |  |  |
| **CA125 (U/mL)** | |  |  |  |  |
|  | **<35** |  |  |  |  |
|  | **≥35** | 0.730 (0.530-1.006) | 0.054 |  |  |
| **Intraoperative management** | | | | |  |
| **Type of surgery** | |  |  |  |  |
|  | **Laparoscopic** |  |  |  |  |
|  | **Laparotomy** | 1.059 (0.916-1.224) | 0.438 |  |  |
| **Blood transfusion** | |  |  |  |  |
|  | **No** |  |  |  |  |
|  | **Yes** | 0.989 (0.802-1.218) | 0.915 |  |  |
| **Primary anastomosis** | | | | |  |
|  | **No** |  |  |  |  |
|  | **Yes** | 1.175 (1.047-1.318) | **0.006** | 1.458 (1.094-1.944) | **0.010** |
| **Perineum tamponade hemostatic** | | | | |  |
|  | **No** |  |  |  |  |
|  | **Yes** | 1.267 (0.644-2.493) | 0.494 |  |  |
| **Postoperative complications** | | | | |  |
| **Obstruction** | |  |  |  |  |
|  | **No** |  |  |  |  |
|  | **Yes** | 0.500 (0.242-1.031) | 0.061 |  |  |
| **Anastomotic fistula** | | | | |  |
|  | **No** |  |  |  |  |
|  | **Yes** | 0.809 (0.527-1.240) | 0.330 |  |  |
| **Operative area infection** | | | | |  |
|  | **No** |  |  |  |  |
|  | **Yes** | 0.778 (0.585-1.034) | 0.084 |  |  |
| **Cardiovascular disease** | | | | |  |
|  | **No** |  |  |  |  |
|  | **Yes** | 0.714 (0.227-2.251) | 0.566 |  |  |
| **Length of stay (days)** | | 1.002 (0.996-1.009) | 0.441 |  |  |
| **Abbreviations: BMI, body mass index (calculated as weight in kilograms divided by height in meters squared); ASA, American Society of Anesthesiologists Physical Status Classification; COPD, chronic obstructive pulmonary disease; CEA, carcino-embryonic antigen; CA19-9; CA12-5, carbohydrate antigen.** | | | | | |
