# Supplementary material for: Perineural invasion affects prognosis of patients undergoing colorectal cancer surgery: a propensity score matching analysis
Source: BMC Cancer. 2023 May 18;23:452. doi: 10.1186/s12885-023-10936-w (PMC10197328; doi:10.1186/s12885-023-10936-w)
Supplement: Supplementary file 8 — Supplementary Material 8 [file 12885_2023_10936_MOESM8_ESM.docx]

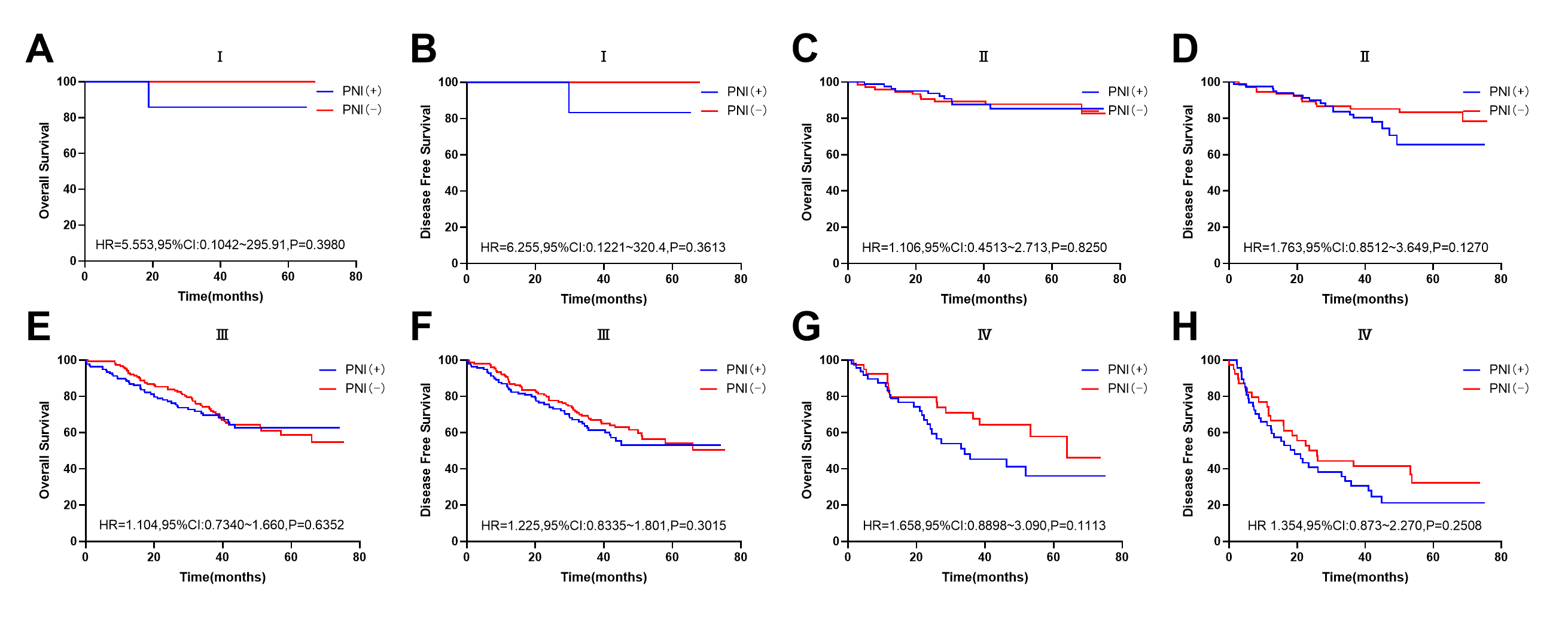


**Supplementary Figure 1: Kaplan-Meier survival curves of OS and DFS for the PNI (+) group at different stages in the matched cohort.** The Kaplan-Meier survival curves of OS of PNI (+) group at Ⅰ(A)、Ⅱ(C)、Ⅲ(E)、Ⅳ(G) stages in the matched cohort. The Kaplan-Meier survival curves of DFS of PNI (+) group at Ⅰ(B)、Ⅱ(D)、Ⅲ(F)、Ⅳ(H) stages in the matched cohort.


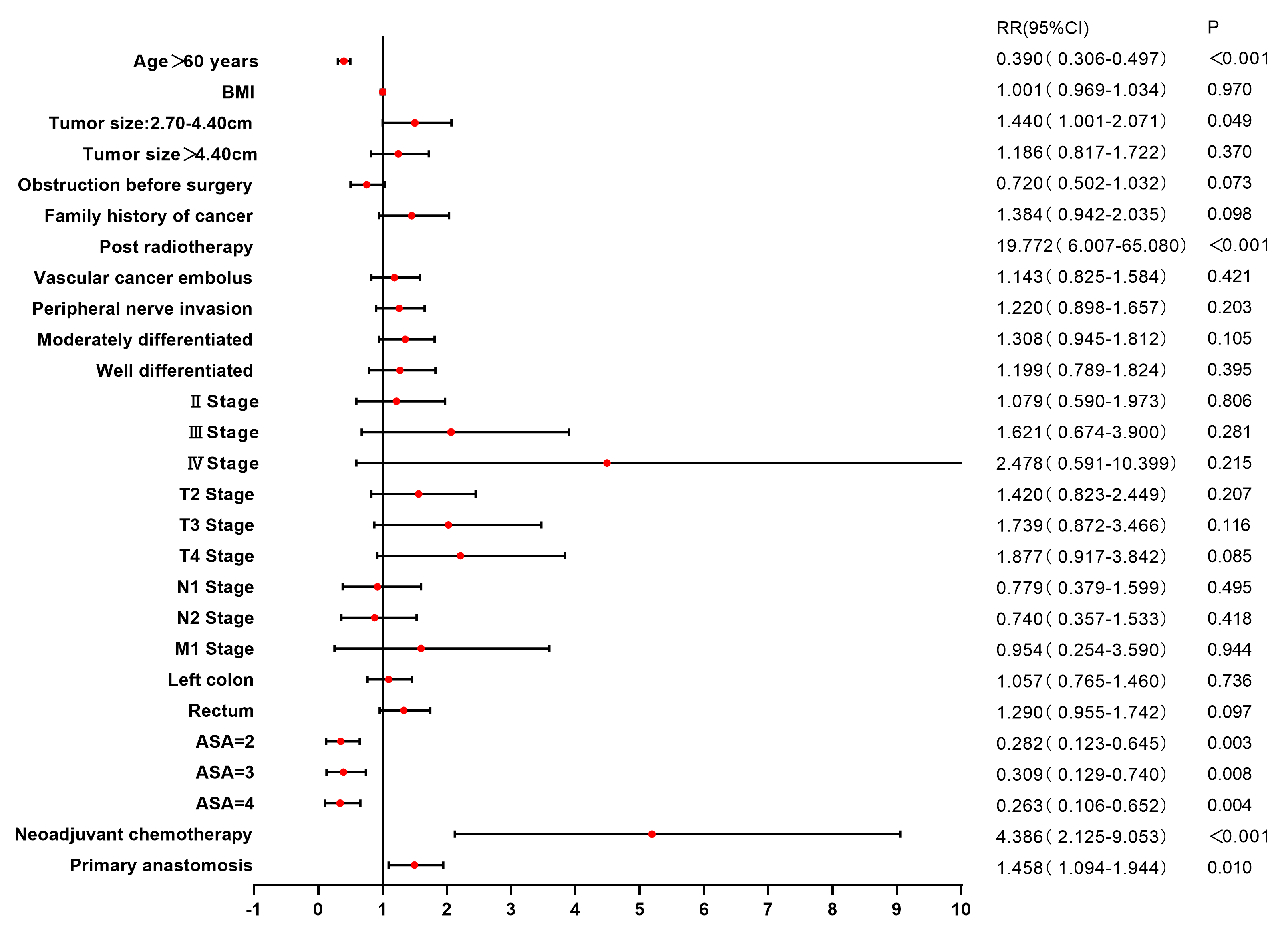


**Supplementary Figure 2: Forest plot of factors associated with postoperative chemotherapy.**


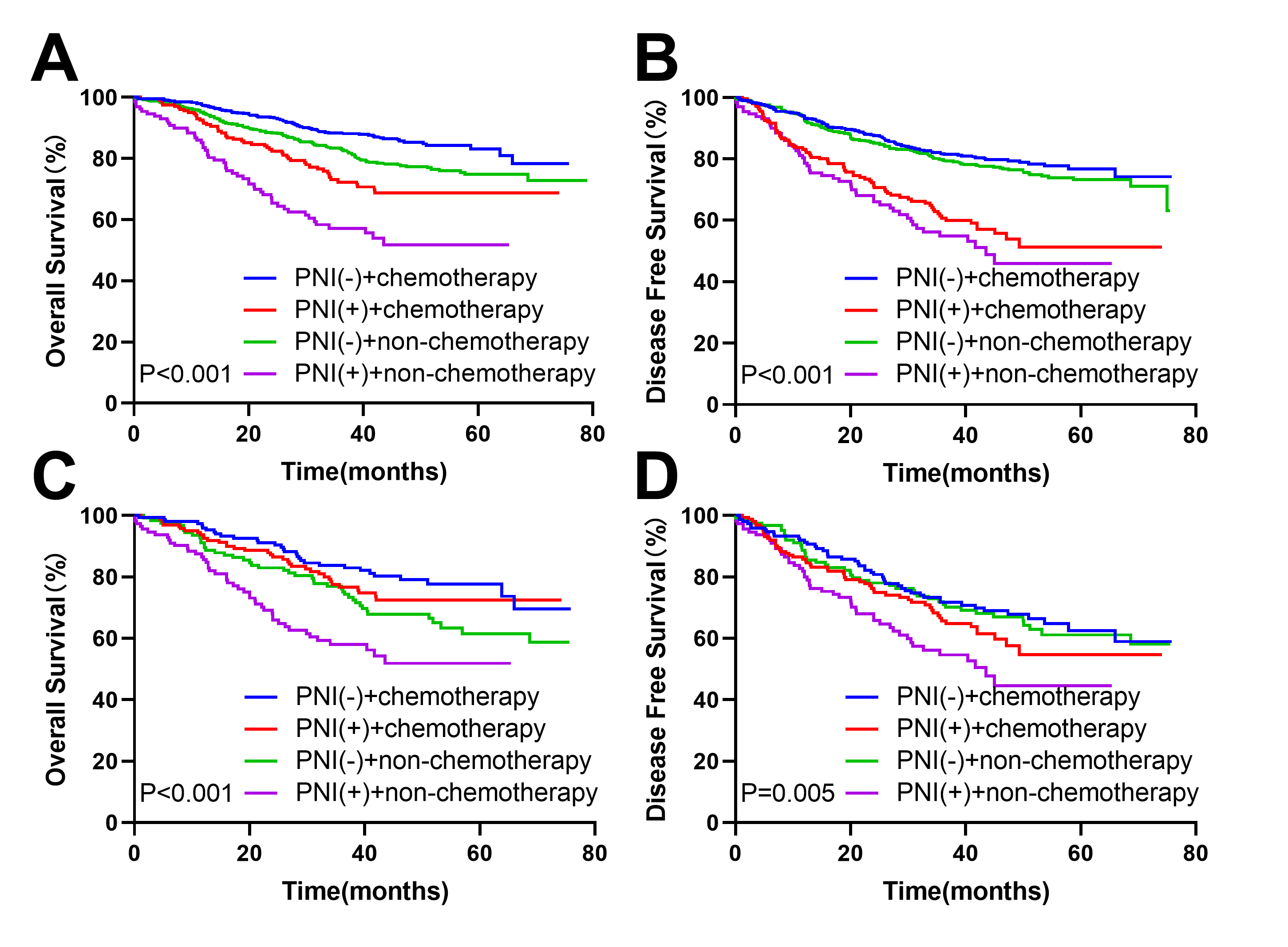


**Supplementary Figure 3: The Kaplan-Meier survival curves of PNI(-) and PNI(+) patients with or without postoperative adjuvant chemotherapy.** The Kaplan-Meier survival curves of OS(A) and DFS(B) for patients in the [original](javascript:;) cohorts. The Kaplan-Meier survival curves of OS(C) and DFS(D) for patients in the matched cohorts.


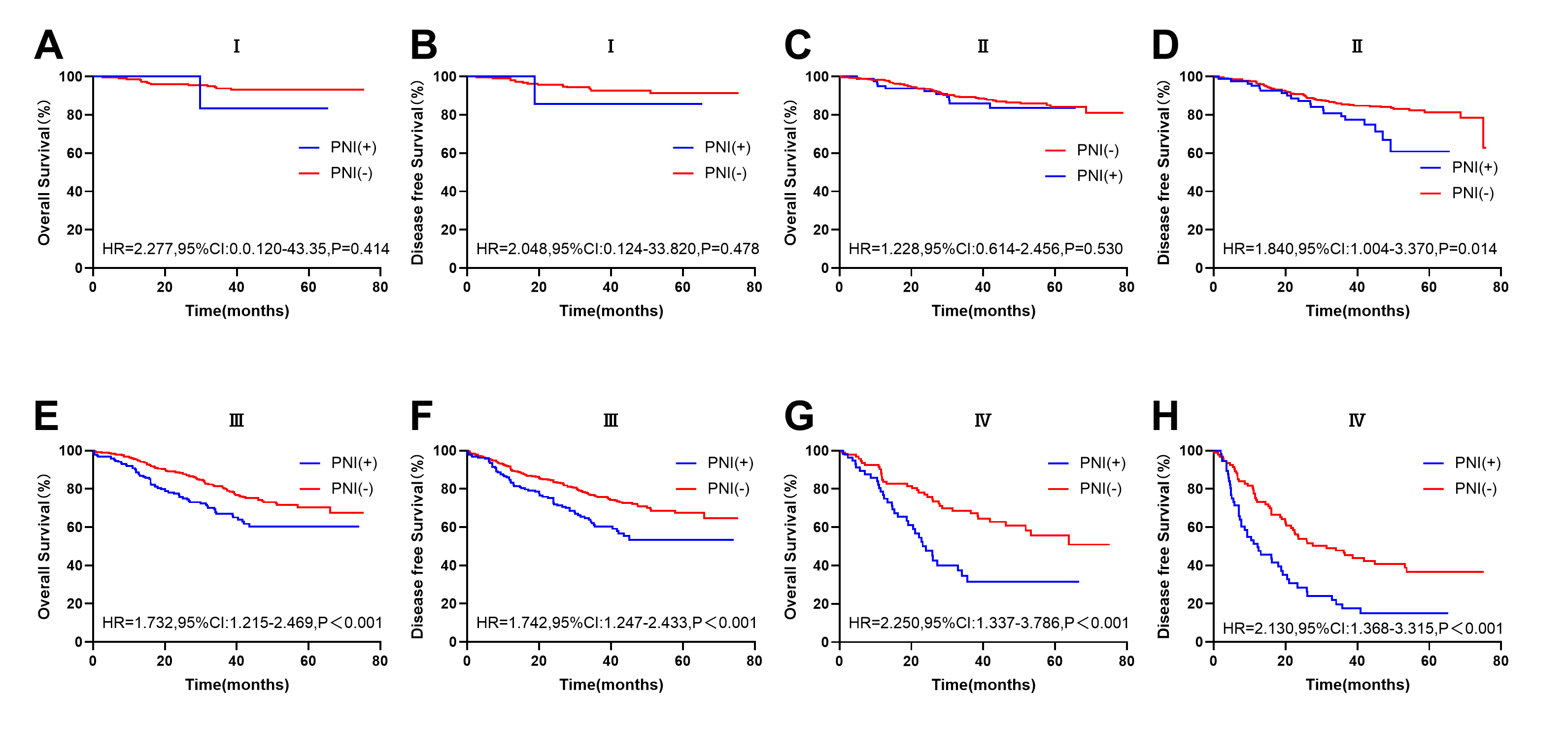


**Supplementary Figure 4: Kaplan-Meier survival curves for different cohorts with different TNM stages.** The Kaplan-Meier survival curves for OS at Ⅰ(A)、Ⅱ(B)、Ⅲ(C)、Ⅳ(D) stages and DFS at Ⅰ(E)、Ⅱ(F)、Ⅲ(G)、Ⅳ(H) stages in the original cohort.
